# Space–time adaptive hierarchical model reduction for parabolic equations

**DOI:** 10.1186/s40323-015-0046-4

**Published:** 2015-10-13

**Authors:** Simona Perotto, Alessandro Zilio

**Affiliations:** MOX, Department of Mathematics, Politecnico di Milano, Piazza Leonardo da Vinci, 32, 20133 Milan, Italy; Centre d’Analyse et de Mathématique Sociales, École des Hautes Études en Sciences Sociales, 190-198 Avenue de France, 75244 Paris Cedex 13, France

**Keywords:** Hierarchical model reduction, Model adaptation, Space–time adaptation, Goal-oriented a posteriori error analysis, Unsteady advection–diffusion–reaction problems

## Abstract

**Background:**

Surrogate solutions and surrogate models for complex problems in many fields of science and engineering represent an important recent research line towards the construction of the best trade-off between modeling reliability and computational efficiency. Among surrogate models, hierarchical model (HiMod) reduction provides an effective approach for phenomena characterized by a dominant direction in their dynamics. HiMod approach obtains 1D models naturally enhanced by the inclusion of the effect of the transverse dynamics.

**Methods:**

HiMod reduction couples a finite element approximation along the mainstream with a locally tunable modal representation of the transverse dynamics. In particular, we focus on the pointwise HiMod reduction strategy, where the modal tuning is performed on each finite element node. We formalize the pointwise HiMod approach in an unsteady setting, by resorting to a model discontinuous in time, continuous and hierarchically reduced in space (c[M($\mathbf{M}$)G(*s*)]-dG(*q*) approximation). The selection of the modal distribution and of the space–time discretization is automatically performed via an adaptive procedure based on an *a posteriori* analysis of the global error. The final outcome of this procedure is a table, named *HiMod lookup diagram*, that sets the time partition and, for each time interval, the corresponding 1D finite element mesh together with the associated modal distribution.

**Results:**

The results of the numerical verification confirm the robustness of the proposed adaptive procedure in terms of accuracy, sensitivity with respect to the goal quantity and the boundary conditions, and the computational saving. Finally, the validation results in the groundwater experimental setting are promising.

**Conclusion:**

The extension of the HiMod reduction to an unsteady framework represents a crucial step with a view to practical engineering applications. Moreover, the results of the validation phase confirm that HiMod approximation is a viable approach.

## Background

The extensive use of scientific computing in many fields of science and engineering requires more and more frequently to reach a compromise between modeling reliability and computational efficiency [1]. This goal is currently pursued in the literature via the set up of two complementary methodologies, i.e., *surrogate solutions* and *surrogate models*. Surrogate solutions are generally formalized with a reduction of the size of the finite dimensional solution, as in the reduced basis approach [2], or in the proper orthogonal decomposition (POD) [3] and proper generalized decomposition (PGD) methods [4, 5].

Surrogate models directly replace the reference model via a simplified formulation as with a geometric multiscale modeling [6, 7] or with compressed sensing [8]. This is usually accomplished by taking advantage of specific features of the problem at hand, such as a prevalent direction in the involved dynamics rather than in the geometry of the computational domain. This is exactly the criterion exploited to settle the hierarchical model (HiMod) reduction proposed in [9, 10]. The HiMod technique derives enriched 1D surrogate models to describe phenomena characterized by a leading dynamics albeit in the presence of locally significant transverse features. In particular, the description properties of purely 1D models are enhanced by keeping track of the transverse dynamics in the reduced model. This is achieved by enriching a finite element discretization of the mainstream with a modal representation of the secondary dynamics. This strategy leads to a 1D finite element model with *ad-hoc* coefficients that implicitly include the generally non-constant description of the transverse dynamics. The possibility of locally tuning the modal expansion to match spatial heterogeneities represents one of the main strengths of the HiMod approach [11].

In this paper, we focus on the pointwise HiMod reduction strategy proposed in [12], where the modal tuning is performed on the finite element nodes. For this reason, the pointwise approach turns out to be the most flexible one among the available HiMod procedures [13], being suited to model both localized and widespread dynamics. In particular, with a view to practical applications, we extend the pointwise HiMod formulation to an unsteady setting by resorting to a discretization discontinuous in time. We generalize the cG(*s*)-dG(*q*) formulation in [14–16] to the HiMod setting, by defining a reduced solution that we denote by c[M($\mathbf{M}$)G(*s*)]-dG(*q*) approximation. We replace the full model with a solution that is continuous in space and discontinuous in time. It is obtained via a Galerkin spatial approximation that combines finite elements of degree *s* with the modal expansion identified by the index $\mathbf{M}$, and via discontinuous piecewise polynomials of degree *q* in time.

The selection of the modal distribution as well as of the space–time discretization represents a crucial step of the HiMod reduction. For this reason, we introduce a preprocessing phase to automatically identify the HiMod solution, for fixed values of *s* and *q*. The final outcome of this phase is a table that identifies the time partition and then, for each time interval, selects the corresponding 1D finite element mesh together with the associated modal distribution. We call this table *HiMod lookup diagram*. To this purpose, we resort to an adaptive procedure based on an *a posteriori* analysis of the global (modeling plus space–time discretization) error. We rely upon a goal-oriented setting [17–19], so that the prediction of the c[M($\mathbf{M}$)G(*s*)]-dG(*q*) model is driven by a physical quantity of interest.

The estimator for the global error consists of a modeling and of a discretization contribution, which are preserved distinct [11, 20–22]. This represents a crucial property with a view to a global adaptation algorithm. In particular, the modeling estimator is a generalization of the goal-oriented hierarchical *a posteriori* error estimator derived in [11] to a time dependent setting, and it includes the temporal discontinuities of the c[M($\mathbf{M}$)G(*s*)]-dG(*q*) scheme. The estimator for the discretization error, in turn, keeps separate the temporal from the spatial contribution [23–26] and it is obtained by including the intrinsic dimensionally hybrid nature of a HiMod approximation into the standard goal-oriented analysis, as in [11].

Although the HiMod lookup diagram is strictly tailored to the problem at hand, we will show that it can be employed to deal with certain variants of such a problem. Thus the computational effort characterizing the preprocessing pays off.

A first validation of the HiMod reduction procedure is also provided in this paper, by dealing with an experimental and modeling study of solute transport in porous media [27].

## The full setting

We introduce the reference parabolic model we aim at reducing via an adaptive space–time model reduction procedure. A standard notation is adopted for the Sobolev spaces associated with the spatial independent variable only, as well as for the space of the functions bounded almost everywhere [28]. Concerning a space–time dependence, we introduce the spaces $L^2(0, T; W)=\big \{ v:(0, T) \rightarrow W :\int _{0}^{T} \Vert v(t) \Vert _W^2 dt< +\infty \big \}$, $H^1(0, T; W)=\big \{ v,\ \frac{\partial v}{\partial t} \in L^2(0, T; W) \big \}$, $C^0([0, T]; W)=\big \{v:[0, T] \rightarrow W\, \text{ continuous } :\forall t \in [0, T], \ \Vert v(t)\Vert _W< +\infty \big \}$, where *W* denotes a generic Hilbert space, with $\Vert \cdot \Vert _W$ the associated norm [29].

### The problem

We select as model to be reduced the unsteady problem

1 $$\begin{aligned} \left\{ \begin{array}{ll} \displaystyle \frac{\partial u}{\partial t}(\mathbf{z}, t) + Lu(\mathbf{z}, t)=f(\mathbf{z}, t) &{} \quad (\mathbf{z}, t)\in Q=\Omega \times I,\\ u(\mathbf{z}, t)=0 &{} \quad (\mathbf{z}, t) \in \partial Q_D=\Gamma _D\times I,\\ D \nabla u(\mathbf{z}, t) \cdot \mathbf{n}=g(\mathbf{z}, t) &{} \quad (\mathbf{z}, t) \in \partial Q_N=\Gamma _N\times I,\\ u(\mathbf{z}, 0)=u_0(\mathbf{z}) &{} \quad \mathbf{z}\in \Omega , \end{array} \right. \end{aligned}$$

where $\Omega \subset \mathbb R^d$ ($d=2, 3$) is the computational domain, $\Gamma _D$ and $\Gamma _N$ constitute a measurable non-overlapping partition of $\partial \Omega$ such that $\partial \Omega =\Gamma _D \cup \Gamma _N$ and ${\mathop{\Gamma}\limits^{\circ}}_D \cap {\mathop{\Gamma}\limits^{\circ}}_N=\emptyset$, $I=(0, T)$ is the time window of interest, and *L* is a generic second-order elliptic operator with diffusive contribution given by $-\nabla \cdot (D \nabla u)$ so that $D \nabla u \cdot \mathbf{n}\equiv \partial _\nu u$ is the conormal derivative of *u*, $\mathbf {n}$ being the unit outward normal vector to $\partial \Omega$. Concerning the data, we choose the source $f \in L^2(0, T; L^2(\Omega ))$, the diffusivity tensor $D=[d_{ij}] \in [L^{\infty }(\Omega )]^{d\times d}$ such that the uniform ellipticity condition holds, the initial datum $u_0\in L^2(\Omega )$, and the Neumann datum $g \in L^2(0, T; L^2(\Gamma _N))$. In the next section, further requirements are added on the computational domain as well as on the boundary conditions in view of the HiMod procedure.

We consider the weak formulation associated with (1), given by: find $u \in V=L^2(0, T; H^1_{\Gamma _D}(\Omega ))\cap H^1(0, T; (H^1_{\Gamma _D}(\Omega ))')$, with $(H^1_{\Gamma _D}(\Omega ))'$ the dual space of $H^1_{\Gamma _D}(\Omega )$, such that

2 $$\begin{aligned} \int _Q \displaystyle \frac{\partial u}{\partial t} v \, d\Omega \, dt + \int _I a\big (u, v \big ) \, dt = \int _Q fv\, d\Omega \, dt + \int _{\partial Q_N} gv \, ds\, dt \quad \forall v\in V, \end{aligned}$$

with $u(\mathbf{x}, 0)=u_0(\mathbf{x})$, and where $a(\cdot , \cdot ):H^1_{\Gamma _D}(\Omega ) \times H^1_{\Gamma _D}(\Omega ) \rightarrow {\mathbb {R}}$ is the bilinear form associated with operator *L*, here assumed continuous and coercive. Problem (2) represents the *full problem*, with *u* the full solution.

The continuous embedding $V \hookrightarrow C^0([0, T]; L^2(\Omega ))$ ensures the temporal continuity to the weak solution *u* in (2).

### The computational domain

Problems suited to a HiMod reduction are defined on domains characterized by a prevalent dimension and the leading dynamics is aligned with such a dimension.

Thus, we assume $\Omega$ to coincide with the *d*-dimensional fiber bundle $\Omega =\bigcup _{x \in \Omega _{1D}} \{ x \} \times \gamma _x$, where $\Omega _{1D}$ is the supporting 1D fiber described by the independent variable *x* and aligned with the dominant dynamics, while $\gamma _x \subset {\mathbb {R}}^{d-1}$ denotes the transverse fiber that is, in general, a function of *x* and parallel to the transverse dynamics. For the sake of simplicity, we assume $\Omega _{1D}\equiv ]x_0,x_1[$ to be rectilinear and we refer to [30] for the more general case of a curved supporting fiber. We partition the boundary $\partial \Omega$ of $\Omega$ into three disjoint sets, $\Gamma _0= \{x_0\}\times \gamma _{x_0}$, $\Gamma _1= \{x_1\}\times \gamma _{x_1}$ and $\Gamma _*=\bigcup _{x\in \Omega _{1D}} \partial \gamma _x$, such that $\partial \Omega = \Gamma _0 \cup \Gamma _1 \cup \Gamma _*$ (see Remark 2 for further details).

**Fig. 1:**
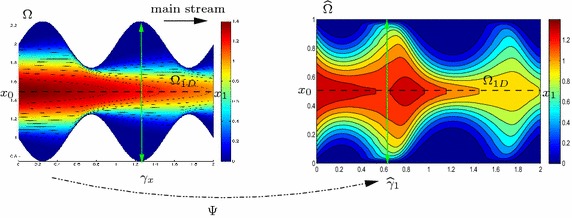
Map $\Psi$ between a 2D sinusoidal domain $\Omega$ and the rectangular reference domain $\widehat{\Omega }$

Now, we map the domain $\Omega$ into a reference bundle $\widehat{\Omega }$, where the computations are easier, free from undetermined constants, and are carried out once and for all. To this aim, for any $x\in \Omega _{1D}$, we introduce the map $\psi _{x}:\gamma _x \rightarrow \widehat{\gamma }_{d-1}$ between the generic fiber $\gamma _x$ and the reference fiber $\widehat{\gamma }_{d-1}\subset {\mathbb {R}}^{d-1}$. Maps $\psi _x$ are instrumental to define the global map $\Psi :\Omega \rightarrow \widehat{\Omega }$, where $\widehat{\Omega }= \bigcup _{x \in \Omega _{1D}} \{ x \}\times \widehat{\gamma }_{d-1}$ denotes the reference computational domain (see Fig. 1 for an example of map $\Psi$). Regularity assumptions are introduced on the maps $\psi _x$ and $\Psi$. In particular, we assume $\psi _x$ to be a $C^1-$diffeomorphism, for all $x\in \Omega _{1D}$, and $\Psi$ to be differentiable with respect to $\mathbf{z}$ (essentially to exclude any kinks along $\Gamma _*$).

We also demand that the supporting fiber $\Omega _{1D}$ is preserved by map $\Psi$, so that the generic point $\mathbf{z} =(x, \mathbf{y})\in \Omega$ is mapped into $\widehat{\mathbf{z}}=\Psi ({\mathbf{z}})=(\widehat{x},\widehat{\mathbf{y}})$, with $\widehat{x}\equiv x$ and $\widehat{\mathbf{y}} = \psi _x (\mathbf{y})$. Finally, without reducing the generality, we assume $\Omega _{1D}$ to be the subset of $\Omega$ with $\mathbf{y}=\mathbf{0}$, i.e., $\Omega _{1D}$ exactly coincides with the centerline of $\Omega$.

#### *Remark 1*

In a 2D setting, we may always select $\psi _x$ as a linear transformation, so that $\widehat{y} = \psi _x (y) = y/L(x)$, with $L(x)=\mathrm {meas} (\gamma _x)$. In 3D a similar choice is possible only for specific configurations, for instance when $\Omega$ is a cylindrical domain. In this case *L*(*x*) coincides with the diameter of the pipe along the centerline.

## HiMod reduction

The HiMod technique has been proposed in [9, 10] with the idea of exploiting the fiber structure demanded on $\Omega$, or, likewise, the preferential dynamics of the phenomenon at hand. Currently, three versions of HiMod reduction have been investigated, from both a theoretical and a numerical viewpoint (see [13] for a survey on the different approaches). Independently of the selected technique, the idea is to manage in a different way the dependence of the solution on the leading and on the transverse dynamics. In particular, since HiMod aims at providing enriched 1D models to be associated with the dominant direction, only the dominant dynamic is discretized via a standard finite element scheme, while getting information on the transverse dynamics via a modal expansion. In this section, we consider two of the available HiMod formulations.

### Uniform HiMod reduction

The distinguishing feature of a uniform HiMod formulation is the adoption of a unique level of detail (i.e., the same number of modal functions) in modeling the transverse dynamics. For the sake of simplicity, we start from a steady setting. The function space associated with a uniform HiMod approach is

3 $$\begin{aligned} V_m= \left\{ v_m(x, \mathbf{y})=\displaystyle \sum _{j=1}^{m} {\widetilde{v}}_j(x) \varphi _j( \psi _x( \mathbf{y} ) ),\quad \text{with }\ {\widetilde{v}}_j\in V_{1D}\right\} , \end{aligned}$$

where $m\in {\mathbb {N}}^+$ is a given integer, $V_{1D}\subseteq H^1(\Omega _{1D})$, and ${\mathcal B}=\{ \varphi _j\}_{j\in {\mathbb {N}}^+}$ is a modal basis of functions in $H^{1}(\widehat{\gamma }_{d-1})$, orthonormal with respect to the $L^2(\widehat{\gamma }_{d-1})$-scalar product. The boundary conditions assigned on $\Gamma _0$ and $\Gamma _1$ are taken into account by the space $V_{1D}$, while the boundary data on $\Gamma _*$ are included in ${\mathcal B}$. Space $V_m$ represents the hierarchy of models. We complete definition (3) by adding a conformity ($V_m \subset V$) and a spectral approximability ($\lim _{m\rightarrow +\infty } \, \inf _{v_m\in V_m} \Vert v - v_m \Vert _V =0$, for any $v\in V$) hypothesis on $V_m$ [9, 10].

#### *Remark 2*

The analysis below is completely general with respect to the boundary data. So far the robustness of the HiMod reduction has been verified when either homogeneous Dirichlet or homogeneous Neumann boundary conditions are assigned on $\Gamma _0$, $\Gamma _1$, $\Gamma _*$, or when non-homogeneous Dirichlet data are enforced on $\Gamma _0$ and $\Gamma _1$. In general, the critical point is the identification of a basis ${\mathcal B}$ matching Robin boundary conditions or non homogeneous data on $\Gamma _*$. A new strategy with respect to this issue has been recently proposed in [31].

With a view to unsteady problems, we introduce a time partition of the time window *I* into *N* subintervals $I_n=(t_{n-1}, t_n]$ of width $k_n=t_n-t_{n-1}$, for $n= 1, \ldots , N$, with $k=\max _n k_n$, $t_0\equiv 0$ and $t_N\equiv T$. This partition induces a subdivision of the cylinder *Q* into *N* space–time slabs $S_n=\Omega \times I_n$, with $n= 1, \ldots , N$. Notice that partition $\{ t_i \}_{i=0}^N$ is not necessarily uniform, to match the possible time heterogeneities of the problem. Now, we look for an approximate solution to (2) coinciding, on each space–time slab $S_n$, with a polynomial of degree at most *q* in time, with $q\in {\mathbb {N}}^+$, and with an element of $V_m$ in space, i.e., a function of the reduced space

4 $$\begin{aligned} V_m^N&=\left\{\vphantom{\left. v_m(x, \mathbf{y}, t)\big |_{I_n}= \displaystyle {\sum \limits _{r=0}^{q}}\ \displaystyle {\sum \limits _{j=1}^{m}} \, t^r\, {\widetilde{v}}^{\, n}_{j, r}(x)\, \varphi _{j, r}( \psi _x( \mathbf{y} ) ),\quad \text{ with }\ {\widetilde{v}}^{\, n}_{j, r}\in V_{1D}\right\}} v_m:(0, T] \rightarrow H^1_{\Gamma _D}(\Omega ) :\; \forall n= 1, \ldots , N\right. \nonumber \\&\quad \quad \left. v_m(x, \mathbf{y}, t)\big |_{I_n}= \displaystyle {\sum \limits _{r=0}^{q}}\ \displaystyle {\sum \limits _{j=1}^{m}} \, t^r\, {\widetilde{v}}^{\, n}_{j, r}(x)\, \varphi _{j, r}( \psi _x( \mathbf{y} ) ),\quad \text{with }\ {\widetilde{v}}^{\, n}_{j, r}\in V_{1D}\right\} . \end{aligned}$$

The boundary conditions in (2) identifies $V_{1D}$ with $H^1_{\gamma _D}(\Omega _{1D})$, where $\gamma _D$ is a subset of $\{0, 1\}$ according to the definition of $\Gamma _D$, while functions $\varphi _{j, r}$ belong to the modal basis ${\mathcal B}$. Moreover, since $0\not \in I_1$, the value $v_m(x, \mathbf{y}, 0)$ has to be specified separately.

**Fig. 2:**
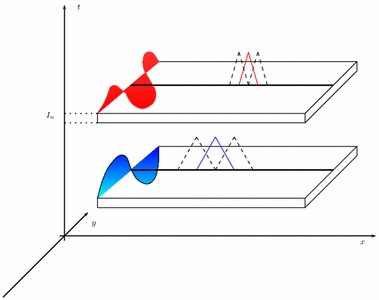
Example of modal distribution and finite element discretization associated with a slabwise uniform HiMod reduction

*A priori*, functions in $V_m^N$ may exhibit a discontinuity at each time level, with continuity from the left. As a consequence, a different number of modal functions can be selected on each time interval $I_n$ (see Fig. 2). This choice leads to replace in (4) the modal index *m* with the index $m_n\in {\mathbb {N}}^+$ with $n=1, \ldots , N$. In such a case we adopt the term space–time *slabwise uniform* HiMod reduction and we change the notation in (4) into $V_\mathbf{m}^N$, where $\mathbf{m}=[m_1, \ldots , m_N]'\in \big [ {\mathbb {N}}^+\big ]^N$ is the vector that collects the number of modes used on each interval $I_n$, with $v_\mathbf{m}$ the generic function in $V_\mathbf{m}^N$.

The possible time discontinuity in $V_\mathbf{m}^N$ leads us to distinguish between the values $v_\mathbf{m}^{n, +}=\lim _{t\rightarrow 0^+}v_\mathbf{m}(x, \mathbf{y}, t_n+t)$ and $v_\mathbf{m}^{n, -}=\lim _{t\rightarrow 0^+}v_\mathbf{m}(x, \mathbf{y}, t_n-t)$, and to define the temporal jump $[v_\mathbf{m}]^n=v_\mathbf{m}^{n, +} - v_\mathbf{m}^{n, -}$ at the generic time $t_n$, for $n=0,\ldots , N-1$. Notice that this jump is identically equal to zero for functions in *V*. This remark allows us to provide a weak formulation for problem (1) equivalent to (2): find $u\in V$ such that

5 $$\begin{aligned} \mathcal A_\mathrm{cGdG}(u, v) = \mathcal F_\mathrm{cGdG}(v)\quad \forall v\in V, \end{aligned}$$

where, for any *w*, $\zeta \in V$,

6 $$\begin{aligned} \mathcal A_\mathrm{cGdG}(w, \zeta )= & {} \displaystyle \sum _{n=1}^{N} \left\{ \ \displaystyle {\int \limits _{S_n}^{}} \dfrac{\partial w}{\partial t} \zeta \, d\Omega \, dt + \displaystyle {\int \limits _{I_n}^{}} a\big ( w, \zeta \big ) \, dt\right\} \nonumber \\&+ \sum _{i=1}^{N-1} \ \int _\Omega [w]^i\, \zeta ^{i, +} \, d \Omega + \int _\Omega w^{0, +} \, \zeta ^{0, +} \, d\Omega \end{aligned}$$

7 $$\begin{aligned} \mathcal F_\mathrm{cGdG}(\zeta )= & {} \displaystyle \int _\Omega w^{0, -} \, \zeta ^{0, +} \, d\Omega + \sum _{n=1}^{N} \left\{ \ \displaystyle {\int \limits _{S_n}^{}} f\zeta \, d\Omega \, dt + \int _{\partial Q_N^n} g\zeta \, ds\, dt\right\} , \end{aligned}$$

with $u^{0, +}=u^{0, -}=u_0(x, \mathbf{y})$ and $\partial {Q}_N^n=\Gamma _N \times I_n$ for $n=1,\ldots , N$. The space–time *slabwise uniform* HiMod formulation can thus be stated: find $u_\mathbf{m}\in V_\mathbf{m}^N$ such that, for any $v_\mathbf{m}\in V_\mathbf{m}^N$,

8 $$\begin{aligned} \mathcal A_\mathrm{cGdG}(u_\mathbf{m}, v_\mathbf{m}) = \mathcal F_\mathrm{cGdG}(v_\mathbf{m}). \end{aligned}$$

The jump terms in (6) provide now an actual contribution, and we distinguish between the HiMod approximation $u_\mathbf{m}^{0, -}\in V_\mathbf{m}^N|_{I_1}$ of the initial datum $u_0$ and $u_\mathbf{m}^{0, +}$ that is unknown. The conformity and the spectral approximability hypotheses are now added slabwise to guarantee the well-posedness of formulation (8). Indeed, due to the discontinuity in time, we can only expect that $V_\mathbf{m}^N \big |_{S_n}\subset V\big |_{S_n}$, while $V_\mathbf{m}^N \not \subset V$.

Concerning the spatial discretization, following [9, 10], we consider a finite element discretization of the function dependence on *x*, after introducing a subdivision, not necessarily uniform, of $\Omega _{1D}$ into subintervals. The time discontinuity allows to employ a different 1D mesh on each space–time slab (see Fig. 2). In particular, we denote by $\mathcal T_{h_n}=\{ K_l^n\}_{l=1}^{{\mathcal M}_n}$ the spatial partition associated with $S_n$ for $n=1, \ldots , N$, with $K_l^n=(x_{l-1}^n, x_l^n)$ the generic subinterval of width $h_l^n=x_l^n-x_{l-1}^n$ for $l=1, \ldots , {\mathcal M}_n$, with $h_n=\max _l h_l^n$ and $x_0^n\equiv x_0$, $x_{{\mathcal M}_n}^n\equiv x_1$. Then, we furnish each $S_n$ with the space $X_{h_n}^{1D, s}$ of the conforming finite elements of degree *s* associated with $\mathcal T_{h_n}$, and with $\dim (X_{h_n}^{1D, s})=N_{h_n}<+\infty$. A standard density hypothesis in $V_{1D}$ is advanced on each finite element space. Thus, the discrete counterpart of formulation (8) is: find $u_\mathbf{m}^h\in V_{\mathbf{m}, h}^N$ such that, for any $v_\mathbf{m}^h\in V_{\mathbf{m}, h}^N$,

9 $$\begin{aligned} \mathcal A_\mathrm{cGdG}(u_\mathbf{m}^h, v_\mathbf{m}^h) = \mathcal F_\mathrm{cGdG}(v_\mathbf{m}^h), \end{aligned}$$

where

10 $$\begin{aligned}&V_{\mathbf{m}, h}^N=\left\{\vphantom{\left. v_\mathbf{m}^h(x, \mathbf{y}, t)\big |_{I_n}= \displaystyle {\sum \limits _{r=0}^{q}}\ \displaystyle {\sum \limits _{j=1}^{m_n}} \, t^r\, {\widetilde{v}}_{j, r}^{\, n, h}(x)\, \varphi _{j, r}( \psi _x( \mathbf{y} ) ),\quad \text{ with }\ {\widetilde{v}}_{j, r}^{\, n, h}\in X_{h_n}^{1D, s}\cap V_{1D}\right\}} v_\mathbf{m}^h:(0, T] \rightarrow H^1_{\Gamma _D}(\Omega ) :\; \forall n= 1, \ldots , N \right. \\&\quad \left. v_\mathbf{m}^h(x, \mathbf{y}, t)\big |_{I_n}= \displaystyle {\sum \limits _{r=0}^{q}}\ \displaystyle {\sum \limits _{j=1}^{m_n}} \, t^r\, {\widetilde{v}}_{j, r}^{\, n, h}(x)\, \varphi _{j, r}( \psi _x( \mathbf{y} ) ),\quad \text{with }\ {\widetilde{v}}_{j, r}^{\, n, h}\in X_{h_n}^{1D, s}\cap V_{1D}\right\} ,\nonumber \end{aligned}$$

$u_\mathbf{m}^{h, 0, -}\in V^N_{\mathbf{m}, h}|_{I_1}$ is a discrete HiMod approximation of $u_0$, and $u_\mathbf{m}^{h, 0, +}$ is an unknown.^1^ It follows $V_{\mathbf{m}, h}^N \subset V_\mathbf{m}^N$, i.e., also the discrete HiMod space $V_{\mathbf{m}, h}^N$ consists of functions continuous in space but discontinuous in time. Notice that, although $V_{\mathbf{m}, h}^N \not \subset V$, in (9) we can extend definitions (6) and (7) to $V_{\mathbf{m}, h}^N$ taking advantage of the slabwise splitting.

By generalizing the notation used in [14–16] to denote finite elements that are continuous in space and discontinuous in time, we refer to $V^N_{\mathbf{m}, h}$ as to the HiMod c[M($\mathbf{m}$)G(*s*)]-dG(*q*) space (and, analogously, to (9) as to the c[M($\mathbf{m}$)G(*s*)]-dG(*q*) HiMod formulation). We mean that, on each $S_n$, the full solution is replaced by a reduced solution continuous in space and discontinuous in time, obtained via a Galerkin approximation based on finite elements of degree *s* combined with the modal expansion associated with the multi-index $\mathbf{m}$ to discretize the space, and piecewise polynomials of degree *q* for the time discretization.

The finite element discretization along $\Omega _{1D}$ allows us to further expand the Fourier coefficient ${\widetilde{v}}_{j, r}^{\, n, h}$ in (10) in terms of the finite element basis $\{ \vartheta _l\}_{l=1}^{N_{h_n}}$ associated with space $X_{h_n}^{1D, s}$, so that any function $v_\mathbf{m}^h \in V_{\mathbf{m},h}^N$ can be represented on the generic time interval $I_n$ as

11 $$\begin{aligned} v_\mathbf{m}^h(x, \mathbf{y}, t)\big |_{I_n}= \displaystyle {\sum \limits _{r=0}^{q}}\ \displaystyle {\sum \limits _{j=1}^{m_n}} \ \displaystyle {\sum \limits _{l=1}^{N_{h_n}}} \, t^r\, {\widetilde{v}}_{j, r, l}^{\, n, h}\, \vartheta _l(x)\, \varphi _{j, r}( \psi _x( \mathbf{y} ) ) \end{aligned}$$

with $n= 1, \ldots , N$. The coefficients ${\widetilde{u}}_{j, r, l}^{\, n, h}$ of $u_\mathbf{m}^h$ become the actual unknowns of the c[M($\mathbf{m}$)G(*s*)]-dG(*q*) HiMod formulation (9).

### HiMod versus PGD

Following the classification proposed in [5], both HiMod reduction and PGD can be categorized as *a priori* approaches since they do not rely on any solution to the problem at hand as, for instance, a POD strategy. Both the methods involve the weak form of the full problem and are based on a classical separation of variables. Nevertheless, while HiMod reduction applies this separation only to the space–time coordinates, PGD involves also problem parameters, such as boundary conditions or material properties, thus increasing the dimension of the space of the unknowns. HiMod applies a different discretization to the variables based on the physics of the problem. The accuracy for each variable may be tuned locally via *a* posteriori arguments. A PGD approach replaces in (3) the known modal function $\varphi _j( \psi _x( \mathbf{y} ) )$, with $\mathbf{y}=(y, z)'$, with a term of the form $F_j(y)G_j(z)$, with $F_j$ and $G_j$ unknowns. The two procedures both lead to 1D algebraic systems. In PGD they are intrinsically nonlinear and, in general, of large dimension. PGD requires therefore specific methods for the nonlinearity. In addition, the construction of the PGD approximation via a successive enrichment of an initial solution closely resembles the heuristic approach initially used in HiMod for selecting the number of transverse modes [10]. In this respect, the automatic selection of the HiMod approximation in [11] may represent an important evolution in a PGD setting as well.

### Pointwise HiMod reduction

A fixed number of modal functions on the whole $\Omega$ may be too restrictive in the presence of spatial heterogeneities. This justifies the formalization of HiMod strategies alternative to the uniform approach, where a different number of modes is adopted in different subdomains of $\Omega$ (via a piecewise HiMod reduction [10, 11]), rather than in correspondence with each finite element node (thanks to a pointwise HiMod formulation [12]). We focus on the last approach. The numerical verification in [12] identifies the pointwise method as the best-performing one in the presence of either widespread or localized transverse dynamics.

The idea exploited in a pointwise HiMod expansion consists in rewriting (11) by emphasizing the sum on the finite element nodes, as

$$\begin{aligned} v_\mathbf{m}^h(x, \mathbf{y}, t)\big |_{I_n}= \displaystyle {\sum \limits _{l=1}^{N_{h_n}}} \, \vartheta _l(x)\, \left[ \, \displaystyle {\sum \limits _{r=0}^{q}}\ \displaystyle {\sum \limits _{j=1}^{m_n}} \, t^r\, {\widetilde{v}}_{j, r, l}^{\, n, h}\, \varphi _{j, r}( \psi _x( \mathbf{y} ) ) \, \right] , \end{aligned}$$

and then in making the modal index $m_n$ dependent on the nodal index *l*. Space $V_{\mathbf{m}, h}^N$ is thus replaced by the new space

12 $$\begin{aligned}&V_{\mathbf{M}, h}^N=\left\{\vphantom{\left. v_\mathbf{M}^h(x, \mathbf{y}, t)\big |_{I_n}=v^h_{\mathbf{M}_n}(x, \mathbf{y}, t)=\displaystyle {\sum \limits _{l=1}^{N_{h_n}}} \vartheta _l(x) \left[ \, \displaystyle {\sum \limits _{r=0}^{q}}\, \displaystyle {\sum \limits _{j=1}^{m_{n, l}}} t^r\, {\widetilde{v}}_{j, r, l}^{\, n, h}\, \varphi _{j, r}( \psi _x( \mathbf{y} ) ) \right] \right\}} v_\mathbf{M}^h:(0, T] \rightarrow H^1_{\Gamma _D}(\Omega ):\; \forall n= 1, \ldots , N \right. \\&\quad \left. v_\mathbf{M}^h(x, \mathbf{y}, t)\big |_{I_n}=v^h_{\mathbf{M}_n}(x, \mathbf{y}, t)=\displaystyle {\sum \limits _{l=1}^{N_{h_n}}} \vartheta _l(x) \left[ \, \displaystyle {\sum \limits _{r=0}^{q}}\, \displaystyle {\sum \limits _{j=1}^{m_{n, l}}} t^r\, {\widetilde{v}}_{j, r, l}^{\, n, h}\, \varphi _{j, r}( \psi _x( \mathbf{y} ) ) \right] \right\} ,\nonumber \end{aligned}$$

where $\mathbf{M}_n=[m_{n, 1}, \ldots , m_{n, N_{h_n}}]'\in \big [ {\mathbb {N}}^+\big ]^{N_{h_n}}$ is the modal nodewise vector collecting the number of modes used at each finite element node of the slab $S_n$ for $n=1,\ldots , N$, whereas $\mathbf{M}$ is just the subindex used to denote a pointwise HiMod approximation. The nodewise tuning of the number of modes leads to an algebraic system sharing the same sparsity pattern as for the uniform case, but with a smaller dimension [12]. The formulation related to space $V_{\mathbf{M}, h}^N$ coincides with a space–time *pointwise* HiMod reduction and will be denoted by c[M($\mathbf{M}$)G(*s*)]-dG(*q*) form. It reads exactly as (9), simply by replacing space $V_{\mathbf{m}, h}^N$ with $V_{\mathbf{M}, h}^N$. Notice that, since definition (12) strictly depends on the finite element discretization, there does not exist a weak counterpart of the pointwise HiMod formulation.

#### Uniform versus pointwise HiMod reduction: an example

We compare the uniform and the pointwise HiMod approaches on the steady test case 4 in [10], where the transport of oxygen in a wavy channel, representing a Bellhouse oxygenator for extra-corporeal circulation [32], is modeled. This problem is characterized by a widespread dynamics, that is suited to be reduced via both the HiMod techniques.

**Fig. 3:**
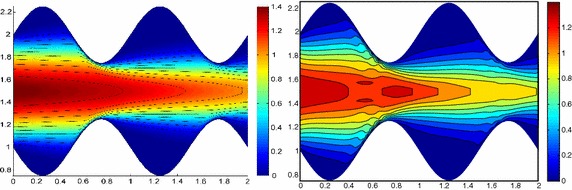
Wavy channel test case: full solution (*left*); uniform HiMod solution for $m=11$ (*right*)

Figure 3, left shows the full solution *u* computed with FreeFem++ [33] on an unstructured uniform mesh of about 50,000 elements and via 2D affine finite elements. The irregular shape of the domain strongly affects the main stream of the flow on the whole domain as highlighted by the bent contour lines.

**Fig. 4:**
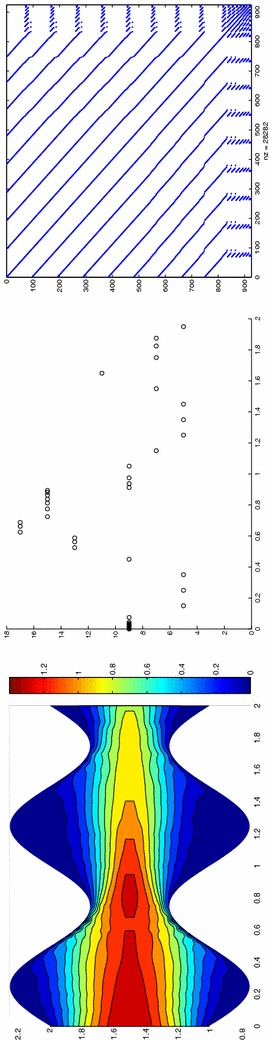
Wavy channel test case: nodewise HiMod solution (*left*) with corresponding modal distribution (*center*) and sparsity pattern (*right*)

As far the HiMod reduction, we discretize the dependence of *u* on *x* via affine finite elements after introducing a partition of uniform step $h =0.1$ on $\Omega _{1D}$. The transverse dynamics are described with a basis ${\mathcal B}$ of sinusoidal functions. To evaluate the integrals of the modal functions, we resort to Gaussian quadrature formulas based on four quadrature nodes per wavelength, at least. No stabilization scheme is used. We first apply the uniform HiMod approach by resorting to 11 modal functions (see Fig. 3, right). Indeed, as shown in [10], at least 11 modes are required to obtain a sufficiently reliable HiMod approximation.

As second assessment, we build the pointwise HiMod approximation $u_\mathbf{M}^h$ associated with the modal distribution $\mathbf{M}$ in Fig. 4, center. By comparing Fig. 3, right with Fig. 4, left we recognize that the two reduced solutions are very similar. In particular, the innermost contour lines associated with $u_\mathbf{M}^h$ are more accurate, despite the lower number of dof involved by the pointwse approximation (48,400 dof characterize $u_{11}^h$ to be compared with 28,282 dof for $u_\mathbf{M}^h$, see the corresponding sparsity pattern in Fig. 4, right).

In accordance with [12], results in Figs. 3 and 4 show the improved modeling capabilities of the pointwise HiMod method vs the uniform approach, for a fixed computational effort. The main issue related to a pointwise formulation is the selection of the nodewise modal distribution. This corroborates the need for an automatic modal selection.

## Adaptive HiMod reduction

Due to its significant impact on practical applications, we consider a goal-oriented framework (see, e.g., [17–19]), so that the predicted reduced model fits a goal functional representing a physical quantity of interest (e.g., mean or pointwise values, fluxes across sections or regions, the energy of the system, the vorticity of a turbulent flow). We denote by *J* the selected functional and we assume it is linear. We aim at approximating, within a prescribed tolerance TOL, the value *J*(*u*), with *u* solution to the full problem (2), via $J(u_\mathbf{M}^h)$, where $u_\mathbf{M}^h$ is the reduced solution identified by a preprocessing phase.

At this stage, we use a uniform and sufficiently fine discretization $\big \{ \big (x_l^n, t_n\big )_{l=1}^{{\mathcal M}_n} \big \}_{n=1}^N$ on $\Omega _{1D} \times I$ so that we can neglect the error due to the space–time discretization.

### The *a posteriori* modeling error analysis

We generalize the error analysis in [11] to an unsteady setting, to automatically produce the HiMod lookup diagram that provides the number of modes to be switched on at each finite element node and at each time of the space–time partition $\big \{ \big (x_l^n, t_n\big )_{l=1}^{{\mathcal M}_n} \big \}_{n=1}^N$ (see Fig. 7, left for an example). The *a posteriori* analysis is carried out on the slabwise uniform HiMod formulation, while the pointwise approximation $u_\mathbf{M}^h$ constitutes the output of the adaptive procedure in the next section.

According to a goal-oriented approach, we introduce the dual problem associated with (8): find $z_\mathbf{m}\in V_\mathbf{m}^N$ such that, for any $v_\mathbf{m}\in V_\mathbf{m}^N$,

13 $$\begin{aligned} \mathcal {A}_\mathrm{cGdG}(v_\mathbf{m}, z_\mathbf{m}) = J_\mathrm{cGdG}(v_\mathbf{m}), \end{aligned}$$

where, for any $\zeta \in V\cup V_\mathbf{m}^N$,

14 $$\begin{aligned} J_\mathrm{cGdG}(\zeta )=\displaystyle {\int \limits _{\Omega }^{}} z_\mathbf{m}^{N, +} \, \zeta ^{N, -} \, d\Omega +\displaystyle \sum _{n=1}^{N} \, \displaystyle {\int \limits _{S_n}^{}} {\widetilde{j}}\, \zeta \, d\Omega \, dt, \end{aligned}$$

where ${\widetilde{j}}$ is the density function associated with the goal functional *J*. Notice that, since $V_\mathbf{m}^N \not \subset V$, *J* has to be defined on $V\cup V_\mathbf{m}^N$ and analogously for $J_\mathrm{cGdG}$. A null final condition, $z_\mathbf{m}^{N, +}=0$, allows to get rid of the first integral in (14), whereas boundary contributions may modify the definition of $J_\mathrm{cGdG}$ when functional *J* involves a control on the boundary. The assignment of boundary conditions to the dual problem is a crucial issue that is usually tackled via the Lagrange identity.

#### *Remark 3*

The bilinear form $\mathcal A_\mathrm{cGdG}(w, \zeta )$ in (6) can be alternatively rewritten integrating by parts the time derivative and recombining the jump terms as

$$\begin{aligned} \displaystyle \sum _{n=1}^{N} \left\{ -\displaystyle {\int \limits _{S_n}^{}} \dfrac{\partial \zeta }{\partial t} w \, d\Omega \, dt + \displaystyle {\int \limits _{I_n}^{}} a\big ( w, \zeta \big ) \, dt \right\} - \sum _{i=1}^{N-1} \ \int _\Omega [\zeta ]^i\, w_m^{i, -} \, d \Omega + \int _\Omega \zeta ^{N, -}\, w^{N, -} \, d \Omega , \end{aligned}$$

for any *w*, $\zeta \in V\cup V_\mathbf{m}^N$. This form better fits the dual setting due to the reverse time scale.

To derive the *a posteriori* modeling error estimator, we need to introduce the enriched primal and dual slabwise uniform HiMod problems,

15 $$\begin{aligned}&\text{ find }\ u_\mathbf{m}^+\in V_{\mathbf{m}^+}^N\quad \text{ s.t. }\quad \mathcal {A}_\mathrm{cGdG}(u_{\mathbf{m}^+}, v_{\mathbf{m}^+}) = {\mathcal F}_\mathrm{cGdG}(v_{\mathbf{m}^+})\quad \forall v_{\mathbf{m}^+}\in V_{\mathbf{m}^+}^N, \end{aligned}$$

16 $$\begin{aligned}&\text{ find }\ z_\mathbf{m}^+\in V_{\mathbf{m}^+}^N\quad \text{ s.t. }\quad \mathcal {A}_\mathrm{cGdG}(v_{\mathbf{m}^+}, z_{\mathbf{m}^+}) = J_\mathrm{cGdG}(v_{\mathbf{m}^+})\quad \forall v_{\mathbf{m}^+}\in V_{\mathbf{m}^+}^N, \end{aligned}$$

with $\mathbf{m}^+>\mathbf{m}$ (i.e., $m_i^+ > m_i$ for $i=1, \ldots , N$). The inclusion $V_{\mathbf{m}}^N\subset V_{\mathbf{m}^+}^N$ guarantees the orthogonality relations

$$\begin{aligned} \mathcal {A}_\mathrm{cGdG}(u_{\mathbf{m}^+}-u_\mathbf{m}, v_\mathbf{m}) = 0, \quad \mathcal {A}_\mathrm{cGdG}(v_\mathbf{m}, z_{\mathbf{m}^+}-z_\mathbf{m}) = 0 \quad \forall v_\mathbf{m} \in V_{\mathbf{m}}^N. \end{aligned}$$

The analysis derived in [11] can be applied to the slabwise uniform HiMod formulations, to state the following

#### **Proposition 1**

*Let*$e_{\mathbf{m}}=u-u_{\mathbf{m}}\in V\cup V_\mathbf{m}^N$*and*$e_{\mathbf{m}^+}=u-u_{\mathbf{m}^+}\in V\cup V_{\mathbf{m}^+}^N$*be the modeling error associated with the reduced formulation* (8) *and* (15), *respectively for*$\mathbf{m}, \mathbf{m}^+\in \big [ {\mathbb {N}}^+\big ]^N$*and with*$\mathbf{m}^+>\mathbf{m}$. *Let us assume that the final dual data*$z_\mathbf{m}^{N, +}$*and*$z_{\mathbf{m}^+}^{N, +}$*are identically equal to zero. Then, if there exists a positive constant*$\sigma _\mathbf{m}<1$*and a modal multi-index*$\mathbf{M}_0\in \big [ {\mathbb {N}}^+ \big ]^N$*such that, for*$\mathbf{m}^+>\mathbf{m}\ge \mathbf{M}_0$,

17 $$\begin{aligned} |J(e_{{\mathbf{m}}^+})| \le \sigma _{\mathbf{m}} \, |J(e_{\mathbf{m}})|, \end{aligned}$$

*the following two-sided inequality holds*

18 $$\begin{aligned} \displaystyle {\frac{|J(\delta u_{{\mathbf{mm}}^+})|}{1+\sigma _{\mathbf{m}}}} \le |J(e_{\mathbf{m}})| \le \displaystyle {\frac{|J(\delta u_{\mathbf{mm}^+})|}{1-\sigma _{\mathbf{m}}}}, \end{aligned}$$

*with*$$\delta u_{\mathbf{mm}^+}=u_{\mathbf{m}^+} - u_\mathbf{m}$$.

Thanks to the requirement on the dual final data, we have that $J_\mathrm{cGdG} \equiv J$. Result (18) identifies the estimator $\eta _{\mathbf{mm}^+}$ for the modeling error $J(e_\mathbf{m})$ with the value $|J(\delta u_{\mathbf{mm}^+})|$, while guaranteeing the efficiency and the reliability of $\eta _{\mathbf{mm}^+}$ via the lower and upper bound, respectively. Following [11], to evaluate estimator $\eta _{\mathbf{mm}^+}$, we can adopt three equivalent formulas, i.e.,

19 $$\begin{aligned} \eta _{\mathbf{mm}^+}={\mathcal A}_\mathrm{cGdG}(\delta u_{\mathbf{mm}^+}, \delta z_{\mathbf{mm}^+})= \rho _p(u_\mathbf{m})(z_{\mathbf{m}^+})=\rho _d(z_{\mathbf{m}})(u_{\mathbf{m}^+}) \end{aligned}$$

with $\delta z_{\mathbf{mm}^+}=z_{\mathbf{m}^+} - z_\mathbf{m}$, and where $\rho _p(u_\mathbf{m})(\cdot )={\mathcal F}_\mathrm{cGdG}(\cdot )-{\mathcal A}_\mathrm{cGdG}(u_\mathbf{m}, \cdot )$, and $\rho _d(z_{\mathbf{m}})(\cdot )=J_\mathrm{cGdG}(\cdot )-{\mathcal A}_\mathrm{cGdG}(\cdot , z_{\mathbf{m}})$ denote the weak primal and dual residual associated with formulation (8) and (13), respectively. Moreover, to make computable $\eta _{\mathbf{mm}^+}$, we replace the reduced primal and dual solutions with corresponding discrete approximations. Estimator $\eta _{\mathbf{mm}^+}$ exhibits the structure typical of a hierarchical error estimator, yet in a goal-oriented framework. We refer to [11] for further computational remarks and for some considerations on hypothesis (17) that represents a generalization of the standard saturation assumption [34–36] to a goal-oriented setting.

### Construction of the HiMod lookup diagram

Estimator $\eta _{\mathbf{mm}^+}$ is now used to automatically select the pointwise HiMod approximation $u_\mathbf{M}^h$ for problem (2) that guarantees the desired accuracy TOL on the functional error $J(u-u_\mathbf{M}^h)$.

**Fig. 5:**
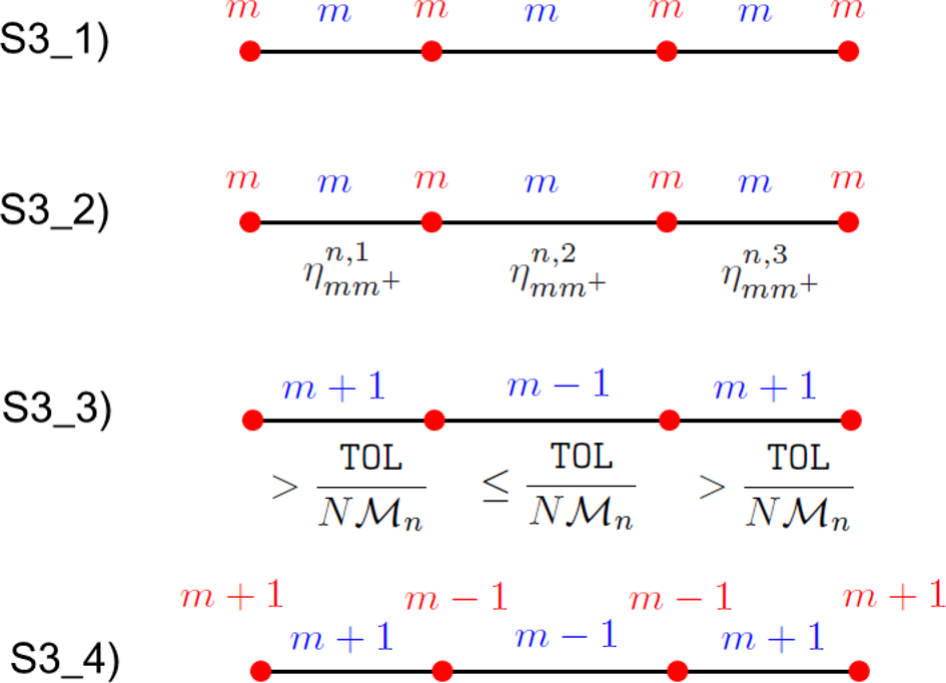
Example of the modal adaptive procedure at stage (S3)

To start the adaptive algorithm, we assign two initial (possibly small) values to the uniform modal indices *m* and $m^+$. Then, we resort to the following five-stage procedure:

1. we compute the discrete uniform reduced primal and dual solutions, $u_m^h$, $u_{m^+}^h$, $z_m$, $z_{m^+}^h$, on the whole space–time cylinder *Q*;
2. we evaluate the modeling estimator $\eta _{mm^+}^n=\eta _{mm^+}\big |_{S_n}$ localized to each space–time slab $S_n$;
3. we apply the adaptive procedure outlined in Fig. 5 on each slab $S_n$ to predict the corresponding nodewise modal distribution $\mathbf{M}_n$, i.e., to build the HiMod lookup diagram (see below for all the details);
4. we compute the discrete pointwise reduced primal and dual solutions, $u_\mathbf{M}^h$, $u_{\mathbf{M}^+}^h$, $z_\mathbf{M}$, $z_{\mathbf{M}^+}^h$, associated with the HiMod diagram yielded at stage (S3);
5. we evaluate the global modeling error estimator $\eta _{\mathbf{MM}^+}$ by employing the pointwise solutions identified at stage (S4). Then, if the global tolerance is met, i.e., $\eta _{\mathbf{MM}^+}\le$TOL, the procedure stops, providing the HiMod lookup diagram in (S3) as final outcome. Vice versa, if $\eta _{\mathbf{MM}^+}>$TOL, we come back to (S2).

Before detailing the adaptive procedure at stage (S3), some remarks are in order.

The computational effort associated with stage (S1) takes advantage of the time discontinuity of the c[M($\mathbf{M}$)G(*s*)]-dG(*q*) scheme. More sophisticated approaches such as checkpointing [37] may be adopted to further reduce the computational costs. The modeling estimator can obviously be evaluated in correspondence with any HiMod approximation [uniform as in (S2), slabwise uniform as in (19), pointwise as in (S5)]. Indeed, via the first definition in (19), it suffices to properly evaluate the bilinear form (6). Concerning the localization of the estimator to $S_n$ at stage (S2), by exploiting again the first definition in (19), we have

20 $$\begin{aligned} \eta _{mm^+}^n&= \mathcal A_\mathrm{cGdG}(\delta u_{mm^+}, \delta z_{mm^+})\big |_{S_n}\\&= \displaystyle {\int \limits _{S_n}^{}} \dfrac{\partial \delta u_{mm^+}}{\partial t} \delta z_{mm^+} \, d\Omega \, dt + \displaystyle {\int \limits _{I_n}^{}} a\big ( \delta u_{mm^+}, \delta z_{mm^+} \big ) \, dt + \displaystyle {\int \limits _{\Omega }^{}} [\delta u_{mm^+}]^{n-1}\, \delta z_{mm^+}^{n-1, +} \, d \Omega .\nonumber \end{aligned}$$

Finally, the HiMod pointwise approximations $u_\mathbf{M}^h$, $z_\mathbf{M}^h$ and $u_{\mathbf{M}^+}^h$, $z_{\mathbf{M}^+}^h$ at stage (S4) are the solutions to problems (5), (13) and (15), (16) settled in the space $V_{\mathbf{M}, h}^N$ and $V_{\mathbf{M}^+, h}^N$, respectively. In particular, we assume that $V_{\mathbf{M}, h}^N$ and $V_{\mathbf{M}^+, h}^N$ share the same spatial partitions $\mathcal T_{h_n}$ for $n=1, \ldots , N$, so that $\mathbf{M}^+$ identifies reduced solutions with a pointwise larger number of modes with respect to $u_\mathbf{M}^h$ and $z_\mathbf{M}^h$.

Let us focus now on the adaptive procedure devised to commute the slabwise evaluations of $\eta _{mm^+}$ into the lookup diagram predicted at stage (S3). We focus on the generic space–time slab $S_n$ and on the case of linear finite elements:

(S3_1) we assign a number of modes equal to *m* to each node and to each subinterval of partition ${\mathcal T}_{h_n}$;
(S3_2) we evaluate the estimator $\eta _{mm^+}^{n, l}=\eta ^n_{mm^+}\big |_{K^n_l}$ localized to each interval $K_l^n$ of ${\mathcal T}_{h_n}$, for $l=1, \ldots , {\mathcal M}_n$;
(S3_3) we invoke an equidistribution criterion on the slabs as well as on the subintervals $K_l^n$. If $\eta _{mm^+}^{n,l}>$TOL$\, \delta _\mathrm{1M}/(N {\mathcal M}_n)$, we increase by one the modal index associated with $K_l^n$ (model refinement); if $\eta _{mm^+}^{n,l}<$TOL$\, \delta _\mathrm{2M}/(N {\mathcal M}_n)$, we decrease by one such an index (model coarsening); otherwise, we preserve the current modal index;
(S3_4) we update the number of modes associated with each finite element node by assigning to the generic node $x_l^n$, for $l=1, \ldots , {\mathcal M}_n -1$, a number of modes equal to $m_{n,l}=\min (m_{K_l^n},m_{K_{l+1}^n})$, with $m_{K_l^n}$ the number of modes assigned on the interval $K^n_l$. In particular, to avoid an abrupt variation of modes on consecutive nodes, the actual value $m_{n,l}^*$ associated with $x_l^n$ coincides with $\max (0.5\, m_{n, l-1}+ 0.5\, m_{n,l +1}-3, m_{n,l})$. The endpoints of $\Omega _{1D}$ are updated separately as $m_{n,0}=m_{K_1^n}$ and $m_{n, {\mathcal M}_n}=m_{K_{{\mathcal M}_n}^n}$ if Dirichlet boundary conditions are not imposed on $\Gamma _0$ and on $\Gamma _1$, respectively. The assignment of the modal indices $m_{n,l}$ predicts the modal multi-index $\mathbf{M}_n=[m_{n, 1}, \ldots , m_{n, N_{h_n}}]'$ for the slab $S_n$.

The procedure in (S3) is exemplified in Fig. 5 for a partition $\mathcal T_{h_n}$ of $\Omega _{1D}$ consisting only of three subintervals $K^n_l$ ($l=1, 2, 3$).

Of course, steps (S3_1)–(S3_4) are replayed on the enriched modal index $m^+$, with a view to the evaluation of the modeling error estimator at stage (S5).

The adaptive modal algorithm includes both model refinement and coarsening. A minimum number of modes constrains the modal coarsening, while a maximum number of adaptive iterations is fixed to avoid too restrictive demands on TOL. The tuning parameters $\delta _\mathrm{1M}$ and $\delta _\mathrm{2M}$ at stage (S3_3) make the adaptive algorithm more robust, while increasing the corresponding computational efficiency. We set $\delta _\mathrm{1M}=0.5$, $\delta _\mathrm{2M}=1.5$. Finally, the modal update at step (S3_4) plays a crucial role since it explains how to build a pointwise HiMod approximation $u_\mathbf{M}^h$ starting from a HiMod lookup diagram, where the modes are associated with the subintervals.

### Numerical verification

The numerical verification is carried out in a 2D setting. Moreover, to select the discrete HiMod space, we choose $q=0$ and $s=1$, i.e., we use linear finite elements to discretize the leading dynamics and functions piecewise constant in time. It can be checked that the adopted time discretization is equivalent to a modified backward Euler scheme [15].

### Reliability of the adaptive HiMod reduction procedure

**Fig. 6:**
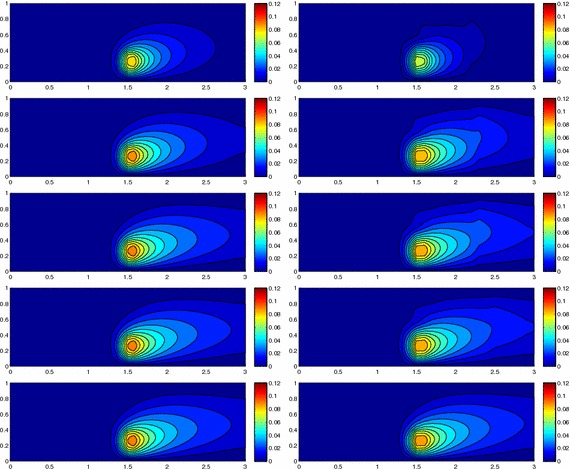
Convection–diffusion of a pollutant, control of $J_{\mathrm{mean}, T}$, modal adaptation: full solution (*left*) and HiMod approximation $u_\mathbf{M}^h$ (*right*), for $t=0.1, 0.2, 0.5, 0.8, 1$ (*top–bottom*)

We approximate problem (1) on the rectangular domain $\Omega =(0, 3)\times (0, 1)$ for $t\in I=(0, 1)$, and by choosing $Lu=-\Delta u + \mathbf{c} \cdot \nabla u$, with $\mathbf{c}=[10, 0]'$. Besides the directionality induced by the advective field, we introduce a local heterogeneity via the source term $f\equiv 10 \chi _{\mathcal D}$, with $\chi _{\mathcal D}$ the characteristic function associated with the elliptic region $\mathcal D=\{ (x, y):(x-1.5)^2 + 4(y-0.25)^2\le 0.01\}$. Concerning the boundary conditions, homogeneous Dirichlet data are assigned on $\partial \Omega \backslash \Gamma _N$, with $\Gamma _N=\{ (3, y):0\le y\le 1\}$, where a homogeneous Neumann datum is enforced. Finally, a null initial datum $u_0$ is chosen. Figure 6, left shows at five different times, the contour plots of the full solution *u* approximated with FreeFem++ via a standard 2D cG(1)-dG(0) scheme on a uniform unstructured mesh of 10252 triangles. As expected, the convective field acts on the purely diffusive phenomenon by horizontally bending the contour lines. From a modeling viewpoint, we are simulating, for instance, the process of convection and diffusion of a pollutant emitted by a chimney localized at $\mathcal D$, in the presence of a moderate horizontal wind. In this context, the full solution *u*(*t*) represents the pollutant concentration in the domain $\Omega$ at a certain time $t\in I$

We aim at controlling the mean value of the full solution on the whole $\Omega$ at the final time $T=1$, i.e., we select the goal functional *J* as $J_{\mathrm{mean}, T}(\zeta )=[ \mathrm {meas}(\Omega ) ]^{-1}\int _{\Omega } \zeta (x, y, 1)\, d\Omega.$ The choice of a localized functional is challenging with a view to the modeling adaptive procedure. The dual problem is characterized by the differential operator $L^*z=-\Delta z - \mathbf{c} \cdot \nabla z$, with source term given by the density function ${\widetilde{j}}(x, y, t)=[ \mathrm {meas}(\Omega ) ]^{-1} \delta _T$ associated with $J_{\mathrm{mean}, T}$, where $\delta _T$ denotes the Dirac distribution associated with the final time. On $\Gamma _N$ a homogeneous Robin boundary condition is imposed, while a homogeneous Dirichlet data is assigned on $\partial \Omega \backslash \Gamma _N$. A null final value $z_\mathbf{m}^{N, +}$ is selected.

Both the primal and dual problems are computed by discretizing the supporting fiber $(0, 3)\times \{ 0.5\}$ via a uniform partition of size $h=0.15$ and the time window with a constant step $k=0.1$. The modal basis ${\mathcal B}$ consists of sinusoidal functions.

Finally, the modeling tolerance TOL is set to $10^{-2}$, while the uniform modal indices *m* and $m^+$ are set to 1 and 3, respectively.

**Fig. 7:**
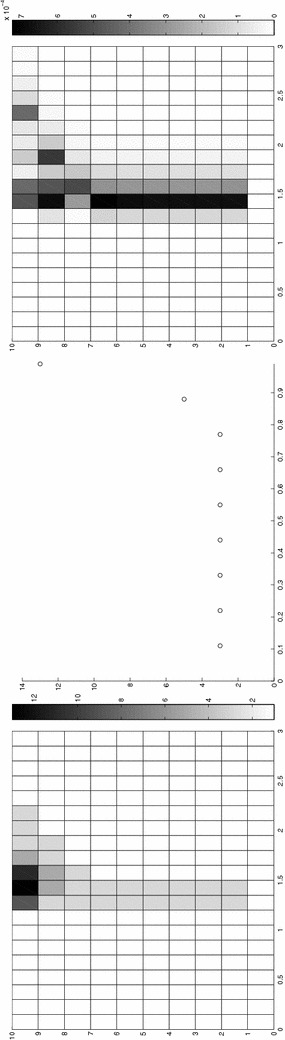
Convection–diffusion of a pollutant, control of $J_{\mathrm{mean}, T}$, modal adaptation: HiMod lookup diagram (*left*); modal distribution at x $=$ 1.5 as a function of time (*center*); space–time distribution of $\eta _{\mathbf{MM}^+}$ (*right*)

The adaptive algorithm converges after 21 iterations and provides as output the HiMod lookup diagram in Fig. 7, left. The diagram coincides with the space–time rectangle $\Omega _{1D}\times I$, where $\Omega _{1D}$ and *I* exhibit the corresponding partition of uniform size *h* and *k*, respectively. A certain number of modal functions is associated with each cell $K_l^n\times k$ for $l=1, \ldots , {\mathcal M}_n$ and $n=1, \ldots , N$. Thus, by resorting to the procedure in Fig. 5, (S3_4) it is possible to build the HiMod pointwise approximation $u_{\mathbf{M}_n}^h$ for $n=1, \ldots , N$, i.e., the reduced solution $u_\mathbf{M}^h$ that guarantees the estimate $|J_{\mathrm{mean}, T}(u)-J_{\mathrm{mean}, T}(u_\mathbf{M}^h)|<$TOL.

The HiMod diagram in Fig. 7, left shows that few modes are demanded on the whole space–time domain, except for the two last time intervals, where a larger number of modes is switched on in correspondence with the localized source and the downstream region. More quantitative information are provided by the plot in Fig. 7, center of the number of modes associated with node $x=1.5$ as a function of time. Only three modes are used on the whole time interval except for the subintervals $I_{N-1}$ and $I_N$, when five and 13 sine functions are required, respectively. The modal distribution predicted by the lookup diagram is completely coherent with a goal-oriented approach. Since we are interested in the mean value of the solution only at the final time, it is reasonable to expect a reliable approximation of the full solution only in correspondence with the last time intervals. This trend is confirmed by the corresponding pointwise HiMod approximation which reproduces more closely the full one during the last times of the simulation (compare Fig. 6, left and right).

In Fig. 7, right we show the value of $\eta _{\mathbf{MM}^+}$ on the same space–time structure of the HiMod diagram. The boxes associated with the largest values of the estimator identify a pattern similar to the one in Fig. 7, left.

### Sensitivity of the adaptive HiMod reduction procedure to the goal-functional

**Fig. 8:**
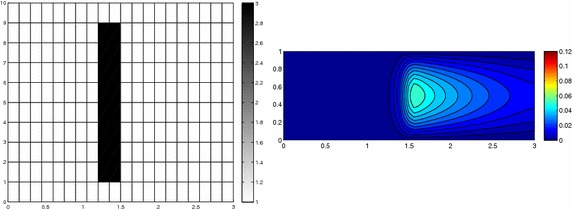
Convection–diffusion of a pollutant, control of $J_{\mathrm{mean}, T}^\mathrm{left}$, modal adaptation: HiMod lookup diagram (*left*); HiMod approximation $u_\mathbf{M}^h$ at $t=T$ (*right*)

We re-run the adaptive procedure by preserving all the input parameters, but for $J=J_{\mathrm{mean}, T}^\mathrm{left}(\zeta )=[ \mathrm {meas}(\Omega ^\mathrm{left}) ]^{-1} \int _{\Omega ^\mathrm{left}} \zeta (x, y, 1)\, d\Omega ^\mathrm{left}$, with $\Omega ^\mathrm{left}=(0, 1.2)\times (0, 1)$. We deal now with a functional localized both in time and space. The adaptive procedure stops after only three iterations by providing the HiMod lookup diagram in Fig. 8, left. A single mode is adopted on the whole time interval in $\Omega ^\mathrm{left}$ where the solution is flat. To ensure tolerance TOL, the modeling error estimator identifies the portion of the domain around $\mathcal D$ as the most problematic one. As a consequence, three sinusoidal functions are used in the two consecutive spatial intervals just before $x=1.5$ for the whole temporal window, except for the last time interval, when a single mode is employed on the entire $\Omega$. The c[M($\mathbf{M}$)G(1)]-dG(0) HiMod approximation at the final time is shown in Fig. 8, right. In agreement with a goal-oriented approach, the reduced solution is far from the full one at *T* in Fig. 6, left-bottom. The mean value is controlled in an area where the full solution is extremely smooth so that a single mode is enough.

### Robustness of the HiMod lookup diagram

**Fig. 9:**
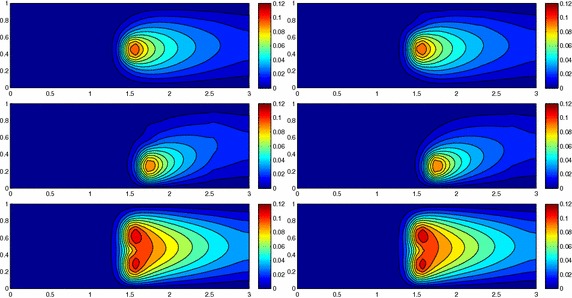
Robustness of the HiMod lookup diagram: HiMod approximations based on the reference lookup diagram (*left*) and on the HiMod adaptive algorithm (*right*) corresponding to the source term $f_1$ (*top*), $f_2$ (*middle*) and $f_3$ (*bottom*)

**Fig. 10:**
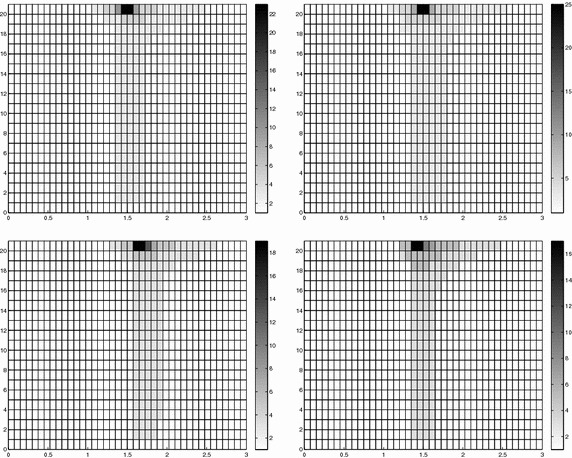
Robustness of the HiMod lookup diagram: reference diagram (*top-left*); diagram corresponding to the source term $f_1$ (*top-right*), $f_2$ (*bottom-left*) and $f_3$ (*bottom-right*)

The computational effort demanded by the adaptive procedure is justified by the possibility to employ the lookup diagram associated with a specific setting to hierarchically reduce a variant of this. Figure 9 performs this check on three variants of the test-case in Fig. 6. In more detail, we adopt the HiMod lookup diagram in Fig. 10, top-left to build the HiMod approximation for three new advection-diffusion problems, characterized by a different choice of the source term, namely, $f_1\equiv 10 \chi _{\mathcal D_1}$ with $\mathcal D_1=\{ (x, y):(x-1.5)^2 + 4(y-0.45)^2\le 0.01\}$ (Fig. 9, top), $f_2\equiv 10 \chi _{\mathcal D_2}$ with $\mathcal D_2=\{ (x, y):(x-1.7)^2 + 4(y-0.25)^2\le 0.01\}$ (Fig. 9, middle) and $f_3\equiv 10 \chi _{\mathcal D_3}$ with $\mathcal D_3=\{ (x, y):(x-1.5)^2 + 4(y-0.25)^2\le 0.01\}\cup \{ (x, y):(x-1.5)^2 + 4(y-0.65)^2\le 0.01\}$ (Fig. 9, bottom), respectively. Figure 9, left shows the HiMod approximations thus obtained. To check the reliability of the obtained solutions, we apply the HiMod adaptive algorithm directly to the three new problems. The corresponding lookup diagrams are gathered in Fig. 10, whereas the associated HiMod approximations are collected in Fig. 9, right. The matching between the contour plots in the two columns of Fig. 9 is substantial, in particular for the two-source test case which represents the most sigificant variant with respect to reference configuration.

**Fig. 11:**
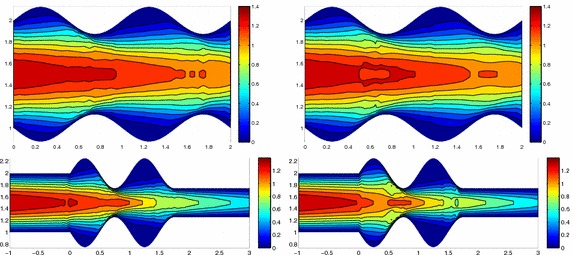
Robustness of the HiMod lookup diagram: HiMod approximations based on the reference lookup diagram (*left*) and on the HiMod adaptive algorithm (*right*) for two possible variants of the original computational domain

As last investigation, we check the robustness of the HiMod lookup diagram with respect to the shape of the computational domain. This is a challenging issue due to the crucial role played by the maps $\psi _x$ and $\Phi$ in the HiMod reduction. For this reason, we focus on a steady setting, in particular on the one in Fig. 3. We consider two variants of the wavy-shaped domain. With the first one, we simply reduce the height of the sinusoidal sections (see Fig. 11, top), whereas in the second variant we add a rectangular channel at the beginning and at the end of the original geometry (see Fig. 11, bottom). Figure 11 compares the solution computed on the modal distribution in Fig. 4, center with the HiMod approximation provided by the adaptive HiMod procedure. As expected, the matching between the two approximations is very good in Fig. 11, top. Despite the abrupt change in the domain shape, we get acceptable results also for the second geometric variant, thus confirming the good robustness of the HiMod lookup diagram with respect to possible changes of the original problem.

## Combined HiMod reduction and space–time adaptation

Goal of this section is to enrich the information provided by the HiMod lookup diagram by predicting also the space–time partition of $\Omega _{1D} \times I$. Consequently, we remove any assumption on the finite element discretization as well as on the time partition $\{I_n\}$. In practice, we expect to replace the diagram in Fig. 7, left with a new diagram characterized by a non uniform horizontal (spatial) and vertical (temporal) spacing.

### The *a posteriori* estimator for the global error

With a view to a global adaptation, following, e.g., [11, 20–22], we derive an *a posteriori* estimator for the global error ${\mathcal E}_\mathbf{m}^h=e_\mathbf{m}+e_\mathbf{m}^h$, where the contributions of the modeling ($e_\mathbf{m}=u-u_\mathbf{m}$) and of the discretization ($e_\mathbf{m}^h=u_\mathbf{m}-u_\mathbf{m}^h$) errors remain distinct. In particular, since we are interested also in an adaptive selection of the space and time step size, we expect that the estimator for the discretization error consists of a spatial contribution separate from the temporal one [23–26].

As for the adaptive HiMod reduction, we carry out the *a posteriori* analysis in a slabwise uniform HiMod setting. The following statement plays a crucial role in the definition of the global error estimator.

#### **Proposition 2**

*We assume that saturation assumption* (17) *holds*, *and we choose*$z_\mathbf{m}^{N, +}=z_{\mathbf{m}^+}^{N, +}=0$. *Then, for any*$\mathbf{m}$, $\mathbf{m}^+\in \big [ {\mathbb {N}}^+ \big ]^N$, *with*$\mathbf{m}^+>\mathbf{m}\ge \mathbf{M}_0$ and $\mathbf{M}_0$*defined as in Proposition*1, *it turns out that*

21 $$\begin{aligned} |J({\mathcal E}_\mathbf{m}^h)|\le \displaystyle \frac{1}{1-\sigma _\mathbf{m}}( |J(\delta u_{\mathbf{mm}^+})| + |J(e_\mathbf{m}^h)| ). \end{aligned}$$

*Moreover, if there exists a constant*$\lambda$*with*$0<\lambda <1$, *such that*

22 $$\begin{aligned} |J(e_\mathbf{m}^h)|\le \lambda |J(e_\mathbf{m})|, \end{aligned}$$

*it additionally holds that*

23 $$\begin{aligned} |J({\mathcal E}_\mathbf{m}^h)|\ge \displaystyle \frac{1-\lambda }{3+\sigma _\mathbf{m}-\lambda }( |J(\delta u_{\mathbf{mm}^+})| + |J(e_\mathbf{m}^h)| ). \end{aligned}$$

#### *Proof*

Estimates (21) and (23) follow from Propositions 3 and 4 in [11], respectively. $\square$

Starting from Proposition 2, we adopt the quantity

24 $$\begin{aligned} \eta _{\mathbf{mm}^+}^h=|J(\delta u_{\mathbf{mm}^+})| + |J(e_\mathbf{m}^h)| \end{aligned}$$

as *a posteriori* error estimator for the global error ${\mathcal E}_\mathbf{m}^h$. As a consequence, inequalities (21) and (23) state the reliability and the efficiency of such an estimator. The first term of $\eta _{\mathbf{mm}^+}^h$ exactly coincides with the modeling error estimator in (19), while the second contribution takes into account the error associated with both the spatial and the temporal discretizations. The main effort of this section will be to explicitly estimate this term, with the additional requirement of distinguishing the space from the time contribution. As in [11], we modify the standard goal-oriented analysis to tackle the intrinsic dimensionally hybrid nature of a HiMod reduced formulation.

Concerning hypothesis (22), it essentially coincides with a sufficient grid resolution requirement since establishing a ratio between the modeling and the discretization errors. With a view to estimate $|J(e_\mathbf{m}^h)|$, we preliminarily prove the following Galerkin orthogonality property for the discretization error $e_\mathbf{m}^h$.

#### **Lemma 1**

*For any*$v_\mathbf{m}^h\in V_{\mathbf{m}, h}^N$, *the following relation holds*

25 $$\begin{aligned} \displaystyle \sum _{n=1}^{N} \left\{ \, \displaystyle {\int \limits _{S_n}^{}} \dfrac{\partial e_\mathbf{m}^h}{\partial t} v_\mathbf{m}^h \, d\Omega \, dt + \displaystyle {\int \limits _{I_n}^{}} a\big ( e_\mathbf{m}^h, v_\mathbf{m}^h \big ) \, dt + \int _\Omega [e_\mathbf{m}^h]^{n-1}\, v_\mathbf{m}^{h, n-1, +} \, d \Omega \right\} =0. \end{aligned}$$

#### *Proof*

We consider the HiMod formulation (8) and the corresponding discrete counterpart (9). The time discontinuity characterizing spaces $V_\mathbf{m}^N$ and $V_{\mathbf{m}, h}^N$ allows us to select the values of $v_\mathbf{m}$ and $v_\mathbf{m}^h$ independently on each $I_n$ for $n=1, \ldots , N$. Thus, we pick both $v_\mathbf{m}$ and $v_\mathbf{m}^h$ to vanish outside $I_n$ so that formulations (8) and (9) reduce to a unique equation on $I_n$: find $u_\mathbf{m}\in V_\mathbf{m}^N\big |_{S_n}$ such that, for any $v_\mathbf{m}\in V_\mathbf{m}^N\big |_{S_n}$,

26 $$\begin{aligned} \mathcal {A}_\mathrm{cGdG}(u_\mathbf{m}, v_\mathbf{m})\big |_{S_n}=\mathcal {F}_\mathrm{cGdG}(v_\mathbf{m})\big |_{S_n}, \end{aligned}$$

and, likewise, find $u_\mathbf{m}^h\in V_{\mathbf{m}, h}^N\big |_{S_n}$ such that, for any $v_\mathbf{m}^h\in V_{\mathbf{m}, h}^N\big |_{S_n}$,

27 $$\begin{aligned} \mathcal {A}_\mathrm{cGdG}(u_\mathbf{m}^h, v_\mathbf{m}^h)\big |_{S_n}=\mathcal {F}_\mathrm{cGdG}(v_\mathbf{m}^h)\big |_{S_n}, \end{aligned}$$

with $\mathcal {A}_\mathrm{cGdG}(w, \zeta )\big |_{S_n}$ defined as in (20) and

$$\begin{aligned} \mathcal {F}_\mathrm{cGdG}(\zeta )\big |_{S_n}= & {} \displaystyle {\int \limits _{S_n}^{}} f \zeta \, d\Omega \, dt + \displaystyle {\int \limits _{\partial {Q}_N^n}^{}} g \zeta \, ds\, dt, \end{aligned}$$

with *w*, $\zeta \in V\cup V_\mathbf{m}^h$. Now, since $V_{\mathbf{m}, h}^N \big |_{S_n}\subset V_\mathbf{m}^N \big |_{S_n}$, we subtract (27) from (26) after identifying $v_\mathbf{m}$ with $v_\mathbf{m}^h$, to get the orthogonality relation

28 $$\begin{aligned} \mathcal {A}_\mathrm{cGdG}(e_\mathbf{m}^h, v_\mathbf{m}^h)\big |_{S_n}=0, \end{aligned}$$

for any $n=1, \ldots , N$. Identity (28) can now be generalized to an arbitrary function $v_\mathbf{m}^h\in V_{\mathbf{m}, h}^N$ by suitably summing through the slabs. This yields identity (25). $\square$

Some notations are now instrumental. Let $R_l^n$ be the region of $\Omega$ defined by $\bigcup _{x\in K_l^n} \{ x \} \times \gamma _x$, with $K_l^n$ the generic subinterval of ${\mathcal T}_{h_n}$, while we denote the interface between $R_\tau ^n$ and $R_{\tau +1}^n$ by $\zeta _\tau ^n$, for $\tau =1, \ldots , {\mathcal M}_n-1$ and $n=1, \ldots , N$, and with $\zeta _0^n\equiv \Gamma _0$ and $\zeta ^n_{{\mathcal M}_n}\equiv \Gamma _1$. Finally, $S_{R_l^n}=R_l^n\times I_n$ denotes the space–time prism associated with $R_l^n,$ while $L_{R_l^n}=\partial R_l^n\times I_n$ identifies the corresponding lateral surface. We introduce now the spatial and temporal local residuals. For a fixed time interval $I_n$ and for any $R_l^n$, we consider the internal residual

29 $$\begin{aligned} r_{R_l^n}=\displaystyle \left( f - \frac{\partial u_\mathbf{m}^h}{\partial t} - L_l^n u_\mathbf{m}^h \right) \Big |_{S_{R_l^n}} \end{aligned}$$

and the boundary residual

$$\begin{aligned} j_{R_l^n} =\left\{ \begin{array}{ll} 0 \quad &{}\text{ on } (\partial R_l^n \cap \Gamma _D) \times I_n\\ 2 (g - \partial _\nu u_\mathbf{m}^h)|_{S_{R_l^n}} &{}\text{ on } (\partial R_l^n \cap \Gamma _N) \times I_n\\ -[\partial _\nu u_\mathbf{m}^h] &{}\text{ on } (\partial R_l^n \cap \mathcal {E}_h^n) \times I_n \end{array} \right. \end{aligned}$$

associated with the discrete HiMod solution $$u_\mathbf{m}^h$$, with $$l=1, \ldots , {\mathcal M}_n$$ and $$n=1, \ldots , N$$, where $$ L_l^n$$ is the restriction of the elliptic operator *L* in (1) to the prism $$S_{R_l^n}$$ and $$[\partial _\nu u_\mathbf{m}^h]$$ is the jump of the conormal derivative of $$u_\mathbf{m}^h$$ across an edge of the skeleton $$\mathcal {E}_h^n=\{ \zeta _\tau ^n \}_{\tau =1}^{{\mathcal M}_n-1}$$. We consider now the temporal residual associated with $$u_\mathbf{m}^h$$ and with the time level $$t_n$$

30 $$\begin{aligned} {\mathcal J}_n = [-u_\mathbf{m}^h]^n = (-u_\mathbf{m}^{h,n,+} + u_\mathbf{m}^{h,n,-}), \end{aligned}$$

together with the initial error

31 $$\begin{aligned} e_\mathbf{m}^{h, 0, -}=u_\mathbf{m}^{0, -}- u_\mathbf{m}^{h, 0, -}. \end{aligned}$$

Finally, we introduce the time projection operator $T_n:V_\mathbf{m}^N\big |_{S_n}\rightarrow H^1_{\Gamma _D}(\Omega )$, for $n=1, \ldots , N$, such that

$$\begin{aligned} T_n v =\displaystyle \frac{1}{k_n}\, \displaystyle {\int \limits _{I_n}^{}} v \, dt\quad \forall v \in V_\mathbf{m}^N\big |_{S_n}, \end{aligned}$$

and the one-dimensional Clément quasi-interpolant ${\mathcal I}^1:L^2(\Omega _{1D})\rightarrow {\mathbb {R}}$ [38]. By definition, the projection error $v-T_n v$ is orthogonal to any function *c* constant in time, so that

32 $$\begin{aligned} \displaystyle \displaystyle {\int \limits _{I_n}^{}} (v - T_n v) c \, dt =0\quad \forall v \in V_\mathbf{m}^N\big |_{S_n}, \end{aligned}$$

whereas the estimate

33 $$\begin{aligned} \Vert v - T_n v\Vert _{L^2(I_n)} \le k_n \Big \Vert \displaystyle \frac{\partial v}{\partial t} \Big \Vert _{L^2(I_n)} \quad \forall v \in V_\mathbf{m}^N\big |_{S_n} \end{aligned}$$

can be proved [16]. Notice that no constant is involved in this result. Concerning the Clément quasi-interpolant, the estimates

34 $$\begin{aligned} \Vert v - {\mathcal I}^1(v) \Vert _{L^2(K)}&\le {\mathcal C}_1 h_K |v|_{H^1(\widetilde{K})}\end{aligned}$$

35 $$\begin{aligned} \Vert v - {\mathcal I}^1(v) \Vert _{L^2(\partial K)}&\le {\mathcal C}_2 h_K^{1/2} \Vert v\Vert _{H^1(\widetilde{K})} \end{aligned}$$

hold, for any $v\in H^1(\Omega _{1D})$, where *K* denotes a generic interval of $\Omega _{1D}$, $\widetilde{K}$ is the associated patch of elements, and with ${\mathcal C}_1$ and ${\mathcal C}_2$ constants depending on the relative size of the elements constituting $\widetilde{K}$ [38].

We are now ready to prove the following result:

#### **Proposition 3**

*Let*$\Omega \subset {\mathbb {R}}^2$. *Let us assume that the approximation*$u_\mathbf{m}^{h, 0, -}$*of the initial datum coincide with the*$L^2$-*projection*${\mathcal P}_{I_1}(u_\mathbf{m}^{0, -})$*of*$u_\mathbf{m}^{0, -}$*onto the space*$V_{\mathbf{m}, h}^N\big |_{I_1}$. *Moreover, we choose*$z_\mathbf{m}^{N, +}=0$. *Then, the following estimate for the functional error*$|J(e_\mathbf{m}^h)|$*holds*

36 $$\begin{aligned} |J(e_\mathbf{m}^h)|\le \displaystyle {\mathcal C} \sum _{n=1}^{N}\, \sum _{l=1}^{{\mathcal M}_n} \left[ \rho ^S_{R_l^n}(u_\mathbf{m}^h)\, \omega ^S_{R_l^n}(z_\mathbf{m} - z_\mathbf{m}^h) + \sum _{i=1}^2 \rho ^{Ti}_{R_l^n}(u_\mathbf{m}^h)\, \omega ^{Ti}_{R_l^n}(z_\mathbf{m} - z_\mathbf{m}^h) \right] , \end{aligned}$$

*with*${\mathcal C}$*a constant depending on the interpolation constants in* (34) and (35), *onqand on*$\max _n m_n$, *where the residuals are defined by*

$$\begin{aligned} \rho _{R_l^n}^S(u_\mathbf{m}^h)&= h_l^n \Vert \overline{r}_{R_l^n} \Vert _{L^2(S_{R_l^n})} + \displaystyle \frac{1}{2} (h_l^n)^{\frac{1}{2}} \Vert \overline{j}_{R_l^n} \Vert _{L^2(L_{R_l^n})}, \\&\quad+ \frac{h_l^n}{k_n^{\frac{1}{2}}} ( \Vert {\mathcal J}_{n-1} \Vert _{L^2(R_l^n)} + \Vert e_\mathbf{m}^{h, 0,-}\Vert _{L^2(R_l^n)} \delta _{1,n}),\\ \rho _{R_l^n}^{T_1}(u_\mathbf{m}^h)&= k_n \Vert r_{R_l^n}- \overline{r}_{R_l^n} \Vert _{L^2(S_{R^n_l})} + k_n^{\frac{1}{2}} ( \Vert {\mathcal J}_{n-1} \Vert _{L^2(R^n_l)} + \Vert e_\mathbf{m}^{h, 0,-}\Vert _{L^2(R_l^n)} \delta _{1,n} ), \\ \rho _{R_l^n}^{T_2}(u_\mathbf{m}^h)&= \displaystyle \frac{ k_n }{2} \Vert j_{R_l^n}- \overline{j}_{R_l^n} \Vert _{L^2(L_{R^n_l})}, \end{aligned}$$

*with*$\overline{r}_{R_l^n}=T_n r_{R_l^n}$, $\overline{j}_{R_l^n}=T_n j_{R_l^n}$, $h_l^n$*and*$k_n$*the length of the generic subinterval*$K_l^n$*and*$I_n$, *respectively for*$l= 1, \ldots , {\mathcal M}_n$*and*$n=1, \ldots , N$, *and with*$\delta _{1,n}$*the Kronecker symbol associated with the first slab*$S_1$, *while the weights are given by*

$$\begin{aligned} \omega _{R_l^n}^S(z_\mathbf{m}-z_\mathbf{m}^h)= & {} \left( \displaystyle \max _{x \in K_l^n} L(x)\right) ^{\frac{1}{2}} \sum _{r=0}^{q}\, \sum _{j=1}^{m_n} \Vert \widetilde{z}_{j,r}^{\, n} - \widetilde{z}_{j,r}^{\, n, h} \Vert _{H^1(\widetilde{K}^n_l)} \Vert t^r\Vert _{L^2(I_n)} \\ \omega _{R_l^n}^{T_1}(z_\mathbf{m}-z_\mathbf{m}^h)= & {} \displaystyle \left\| \frac{\partial (z_\mathbf{m}-z_\mathbf{m}^h)}{\partial t} \right\| _{L^2(S_{R_{l}^n})},\ \omega _{R_l^n}^{T_2}(z_\mathbf{m}-z_\mathbf{m}^h) = \left\| \frac{\partial (z_\mathbf{m}-z_\mathbf{m}^h)}{\partial t} \right\| _{L^2(L_{R_{l}^n})}, \end{aligned}$$

*with*

37 $$\begin{aligned} \widetilde{K}^n_l=\left\{ \begin{array}{ll} K^n_1\cup K^n_2, &{}\quad \text{ for }\ l=1,\\ K^n_{l-1}\cup K^n_l\cup K^n_{l+1} &{}\quad \text{ for }\ l= 2, \ldots , {\mathcal M}_n-1\\ K^n_{{\mathcal M}_n-1}\cup K^n_{{\mathcal M}_n}&{}\quad \text{ for }\ l={\mathcal M}_n, \end{array} \right. \end{aligned}$$

*the patch associated with the subinterval*$K^n_l$, $L(x)=\mathrm {meas} (\gamma _x)$, $\widetilde{z}_{j,r}^{\, n}$*and*$\widetilde{z}_{j,r}^{\, n, h}$*the modal coefficients associated with the dual solution*$z_{\mathbf{m}}$*and with the corresponding discretization*$z_\mathbf{m}^{h}$, *respectively*.

#### *Proof*

We start from the dual problem (13) by choosing $v_\mathbf{m}=e_\mathbf{m}^h$ and we apply the orthogonality relation (25). It follows that, for any $v_\mathbf{m}^h\in V_{\mathbf{m}, h}^N$,

$$\begin{aligned} \big | J(e_\mathbf{m}^h) \big |&= \big | \mathcal {A}_\mathrm{cGdG}(e_\mathbf{m}^h, z_\mathbf{m}) \big |\\&= \left| \displaystyle \sum _{n=1}^N \left\{ \int _{S_n} \frac{\partial e_\mathbf{m}^h}{\partial t} (z_\mathbf{m}-v_\mathbf{m}^h) \, d \Omega d t + \int _{I_n} a(e_\mathbf{m}^h, z_\mathbf{m} -v_\mathbf{m}^h) \, dt\right. \right. \\&- \left. \left. \int _{\Omega } [e_\mathbf{m}^h]^{n-1} v_\mathbf{m}^{\, h, n-1, +}\, d\Omega \right\} + \sum _{i=1}^{N-1} \int _\Omega [e_\mathbf{m}^h]^i z_\mathbf{m}^{i,+} \, d \Omega + \int _\Omega e_\mathbf{m}^{\, h, 0,+} z_\mathbf{m}^{0,+} \, d \Omega \right| . \end{aligned}$$

The identification of $J_\mathrm{cGdG}(e_\mathbf{m}^h)$ with $J(e_\mathbf{m}^h)$ follows from the requirement on the dual final datum. We add and subtract the value $\int _\Omega e_\mathbf{m}^{h, 0, -} \big ( z_\mathbf{m} - v_\mathbf{m}^h\big )^{0, +}d\Omega$, by exploiting in (31) the choice $u_\mathbf{m}^{h, 0, -}={\mathcal P}_{I_1}(u_\mathbf{m}^{0, -})$ for the primal initial datum. A manipulation of the jump contributions combined with the definition of projection operator yields

$$\begin{aligned} \big | J(e_\mathbf{m}^h) \big |&= \left|\vphantom{\left. \left. \int _{\Omega } [e_\mathbf{m}^h]^{n-1} (z_\mathbf{m} - v_\mathbf{m}^h)^{\, n-1, +}\, d\Omega \right\} + \int _{\Omega } e_\mathbf{m}^{h, 0, -} \big ( z_\mathbf{m} - v_\mathbf{m}^h\big )^{0, +}d\Omega \right|} \displaystyle \sum _{n=1}^N \left\{ \int _{S_n} \frac{\partial e_\mathbf{m}^h}{\partial t} (z_\mathbf{m}-v_\mathbf{m}^h) \, d \Omega d t + \int _{I_n} a(e_\mathbf{m}^h, z_\mathbf{m} -v_\mathbf{m}^h) \, dt \right. \right. \\&\quad+\left. \left. \int _{\Omega } [e_\mathbf{m}^h]^{n-1} (z_\mathbf{m} - v_\mathbf{m}^h)^{\, n-1, +}\, d\Omega \right\} + \int _{\Omega } e_\mathbf{m}^{h, 0, -} \big ( z_\mathbf{m} - v_\mathbf{m}^h\big )^{0, +}d\Omega \right| . \end{aligned}$$

After exploiting relation (26) with $v_\mathbf{m}=z_\mathbf{m} - v_\mathbf{m}^h$, we integrate by parts on the regions $R_l^n$:

$$\begin{aligned} \big | J(e_\mathbf{m}^h) \big |&= \left|\vphantom{\left. \left. \int _{R_l^n} [-u_\mathbf{m}^h]^{n-1} (z_\mathbf{m} - v_\mathbf{m}^h)^{n-1, +}\, d R_l^n\right\} + \sum _{\widetilde{l}=1}^{M_1} \int _{R^1_{\widetilde{l}}} e_\mathbf{m}^{h, 0, -} \big ( z_\mathbf{m} - v_\mathbf{m}^h\big )^{0, +}dR^1_{\widetilde{l}} \right|} \displaystyle \sum _{n=1}^N \, \sum _{l=1}^{{\mathcal M}_n} \left\{ \int _{I_n} \left[ \int _{R_l^n} \Big ( f - \frac{\partial u_\mathbf{m}^h}{\partial t} - L_l^n u_\mathbf{m}^h \Big ) (z_\mathbf{m}-v_\mathbf{m}^h) \, d R_l^n \right. \right. \right. \\&\quad+ \left. \int _{\partial R_l^n \cap \Gamma _N} g (z_\mathbf{m}-v_\mathbf{m}^h) \, ds - \int _{\partial R_l^n} \partial _\nu u_\mathbf{m}^h (z_\mathbf{m}-v_\mathbf{m}^h) \, ds\right] \, dt \\&\quad+\left. \left. \int _{R_l^n} [-u_\mathbf{m}^h]^{n-1} (z_\mathbf{m} - v_\mathbf{m}^h)^{n-1, +}\, d R_l^n\right\} + \sum _{\widetilde{l}=1}^{M_1} \int _{R^1_{\widetilde{l}}} e_\mathbf{m}^{h, 0, -} \big ( z_\mathbf{m} - v_\mathbf{m}^h\big )^{0, +}dR^1_{\widetilde{l}} \right| . \end{aligned}$$

Thanks to definitions (29) and (30), we have

38 $$\begin{aligned} \big | J(e_\mathbf{m}^h) \big |&\le \displaystyle \sum _{n=1}^N \, \sum _{l=1}^{{\mathcal M}_n} \left\{ \underbrace{\left| \int _{S_{R_l^n}} r_{R_l^n} (z_\mathbf{m}-v_\mathbf{m}^h) \, d{R_l^n}dt \right| }_\mathrm{(I)} \right. \nonumber \\&\quad+\left. \underbrace{\frac{1}{2}\left| \int _{L_{R_l^n}} j_{R_l^n} (z_\mathbf{m}-v_\mathbf{m}^h) \, dsdt\right| }_\mathrm{(II)} + \underbrace{\left| \int _{R_l^n} {\mathcal J}_{n-1} (z_\mathbf{m}-v_\mathbf{m}^h)^{n-1, +} \, d R_l^n \right| }_\mathrm{(III)}\right\} \nonumber \\&\quad+ \sum _{\widetilde{l}=1}^{M_1} \underbrace{\left| \int _{R^1_{\widetilde{l}}} e_\mathbf{m}^{h, 0, -} \big ( z_\mathbf{m} - v_\mathbf{m}^h\big )^{0, +}dR^1_{\widetilde{l}} \right| }_\mathrm{(IV)}. \end{aligned}$$

We consider separately the four terms (I)–(IV). In particular, we choose $v_\mathbf{m}^h$ coinciding with $z_\mathbf{m}^h + T_n({\mathcal I}^1(z_\mathbf{m}-z_\mathbf{m}^h))$, with $z_\mathbf{m}^h$ the discrete HiMod approximation of the dual solution. In particular, the Clément operator involves only the *x*-dependent modal coefficients since it is one-dimensional. Notice that, since we estimate slabwise the terms (I)–(IV), all the functions in $V_\mathbf{m}^N$ and $V_{\mathbf{m}, h}^N$ have to be meant restricted to $I_n$, for each $n=1, \ldots , N$. Function $v_\mathbf{m}^h$ is extended to zero outside $I_n$ when considered as a function of $V_{\mathbf{m}, h}^N$.

To exploit the projection and the interpolation estimates in (33)–(35), we consider the following splitting

39 $$\begin{aligned} z_\mathbf{m}-v_\mathbf{m}^h=[(z_\mathbf{m} - z_\mathbf{m}^h) - T_n(z_\mathbf{m} - z_\mathbf{m}^h) + T_n(z_\mathbf{m} - z_\mathbf{m}^h) - T_n({\mathcal I}^1(z_\mathbf{m}-z_\mathbf{m}^h))]. \end{aligned}$$

Let us focus on term (I). Using the splitting above, the definition of the averaged residual $\overline{r}_{R_l^n}$ and of the projection operation $T_n$, and by combining results (32) and (33) with the Cauchy–Schwarz inequality, we obtain

$$\begin{aligned} \mathrm{(I)}&= \left| \int _{S_{R_l^n}} \big ( r_{R_l^n} - \overline{r}_{R_l^n} \big )\big [ z_\mathbf{m} - z_\mathbf{m}^h - T_n(z_\mathbf{m} - z_\mathbf{m}^h)\big ] \, d{R_l^n}dt \right. \\&\quad+ \left. \int _{R_l^n} \left[ T_n\big (z_\mathbf{m} - z_\mathbf{m}^h - {\mathcal I}^1(z_\mathbf{m}-z_\mathbf{m}^h)\big )\int _{I_n}r_{R_l^n} \, dt\right] \, dR_l^n\right| \\&\le \int _{R_l^n} \Vert r_{R_l^n} - \overline{r}_{R_l^n} \Vert _{L^2(I_n)}\Vert z_\mathbf{m} - z_\mathbf{m}^h - T_n(z_\mathbf{m} - z_\mathbf{m}^h) \Vert _{L^2(I_n)}\, d{R_l^n}\\&\quad+\left| \int _{S_{R_l^n}} \overline{r}_{R_l^n} \big (z_\mathbf{m} - z_\mathbf{m}^h - {\mathcal I}^1(z_\mathbf{m} -z_\mathbf{m}^h)\big )\, d{R_l^n}dt \right| \\&\le k_n \Vert r_{R_l^n} - \overline{r}_{R_l^n} \Vert _{L^2(S_{R_l^n})} \left\| \frac{\partial (z_\mathbf{m} - z_\mathbf{m}^h)}{\partial t} \right\| _{L^2(S_{R_l^n})}\\&\quad+ \Vert \overline{r}_{R_l^n} \Vert _{L^2(S_{R_l^n})}\Vert (z_\mathbf{m} - z_\mathbf{m}^h - {\mathcal I}^1(z_\mathbf{m}-z_\mathbf{m}^h)\Vert _{L^2(S_{R_l^n})}. \end{aligned}$$

We now consider separately the norm associated with the interpolation error. Let $w_\mathbf{m}$ be a generic element in $V_\mathbf{m}^N$. By exploiting the modal expansion for $w_\mathbf{m}$ and the orthonormality of the modal basis, together with interpolation estimate (34), we obtain

40 $$\begin{aligned}&\Vert w_\mathbf{m} -{\mathcal I}^1(w_\mathbf{m})\Vert ^2_{L^2(S_{R_l^n})}\nonumber \\&\quad =\displaystyle \int _{S_{R_l^n}} \left\{ \displaystyle {\sum \limits _{r=0}^{q}}\ \displaystyle {\sum \limits _{j=1}^{m_n}} t^r \varphi _{j, r}( \psi _x( { y} ) ) \big [ {\widetilde{w}}_{j, r}^{\, n}-{\mathcal I}^1({\widetilde{w}}_{j, r}^{\, n})\big ](x) \, \right\} ^2\, dR_l^ndt\nonumber \\&\quad =\displaystyle {\sum \limits _{r=0}^{q}}\ \displaystyle {\sum \limits _{j=1}^{m_n}} \displaystyle \int _{{\mathcal I}_n} t^{2r} \int _{K_l^n} \left[ \int _{\widehat{\gamma }_1} \varphi ^2_{j, r}(\widehat{y}) \big | {\mathcal D}^{-1}(x, \psi _x^{-1}(\widehat{y}))\big | \, d\widehat{y} \right] \big [ {\widetilde{w}}_{j, r}^{\, n}(x)-{\mathcal I}^1({\widetilde{w}}_{j, r}^{\, n})(x) \big ]^2\, dK_l^ndt\nonumber \\&\quad =\displaystyle {\sum \limits _{r=0}^{q}}\ \displaystyle {\sum \limits _{j=1}^{m_n}} \displaystyle \int _{I_n} t^{2r} \max _{x \in K_l^n} L(x) \Vert {\widetilde{w}}_{j, r}^{\, n}-{\mathcal I}^1({\widetilde{w}}_{j, r}^{\, n}) \Vert ^2_{L^2(K_l^n)}dt\nonumber \\&\quad \le {\mathcal C}_1^2 \max _{x \in K_l^n} L(x)\, (h_l^n)^2 \displaystyle {\sum \limits _{r=0}^{q}}\ \displaystyle {\sum \limits _{j=1}^{m_n}} | {\widetilde{w}}_{j, r}^{\, n} |^2_{H^1(\widetilde{K}_l^n)} \Vert t^r\Vert ^2_{L^2(I_n)}, \end{aligned}$$

where ${\mathcal D}(x, \psi _x^{-1}(\widehat{y}))=L(x)^{-1}$ denotes the Jacobian associated with the map $\psi _x$, and with $\widehat{\gamma }_1$ the reference fiber for the two-dimensional setting. Via this estimate, we obtain the following bound for the term (I) in (38):

41 $$\begin{aligned} \mathrm{(I)}&\le k_n \Vert r_{R_l^n} - \overline{r}_{R_l^n} \Vert _{L^2(S_{R_l^n})} \left\| \frac{\partial (z_\mathbf{m} - z_\mathbf{m}^h)}{\partial t} \right\| _{L^2(S_{R_l^n})}\\&\quad+ {\mathcal C} \Vert \overline{r}_{R_l^n} \Vert _{L^2(S_{R_l^n})} h_l^n \left( \displaystyle \max _{x \in K_l^n} L(x)\right) ^{\frac{1}{2}} \sum _{r=0}^{q}\, \sum _{j=1}^{m_n} | \widetilde{z}_{j,r}^{\, n} - \widetilde{z}_{j,r}^{\, n, h} |_{H^1(\widetilde{K}^n_l)} \Vert t^r\Vert _{L^2(I_n)},\nonumber \end{aligned}$$

with $\mathcal C$ a constant depending on ${\mathcal C}_1$ in (34), *q* and $m_n$. From now on, $\mathcal C$ denotes a constant whose value may change from line to line. Term $\mathrm{(II)}$ can be bounded analogously to contribution $\mathrm{(I)}$, by restricting the computations on the lateral surface $L_{R_l^n}$ of $S_{R_l^n}$. This yields

42 $$\begin{aligned} \mathrm{(II)}\le & {} \displaystyle \frac{k_n}{2} \Vert j_{R_l^n} - \overline{j}_{R_l^n} \Vert _{L^2(L_{R_l^n})} \left\| \frac{\partial (z_\mathbf{m} - z_\mathbf{m}^h)}{\partial t} \right\| _{L^2(L_{R_l^n})}\nonumber \\&+ \displaystyle \frac{1}{2} \Vert \overline{j}_{R_l^n} \Vert _{L^2(L_{R_l^n})}\Vert (z_\mathbf{m} - z_\mathbf{m}^h - {\mathcal I}^1(z_\mathbf{m}-z_\mathbf{m}^h)\Vert _{L^2(L_{R_l^n})}. \end{aligned}$$

Inequality (40) is replaced by a corresponding trace estimate, obtained essentially by invoking result (35) instead of (34), to have

43 $$\begin{aligned} \Vert w_\mathbf{m} -{\mathcal I}^1(w_\mathbf{m})\Vert ^2_{L^2(L_{R_l^n})} \le {\mathcal C}_2^2 \max _{x \in K_l^n} L(x)\, h_l^n \displaystyle {\sum \limits _{r=0}^{q}}\ \displaystyle {\sum \limits _{j=1}^{m_n}} \Vert {\widetilde{w}}_{j, r}^{\, n} \Vert ^2_{H^1(\widetilde{K}_l^n)} \Vert t^r\Vert ^2_{L^2(I_n)}, \end{aligned}$$

for any $w_\mathbf{m}\in V_\mathbf{m}^N$. Combining this result with (42), we attain the following control for the second term in (38):

$$\begin{aligned} \mathrm{(II)}&\le \displaystyle \frac{k_n}{2} \Vert j_{R_l^n} - \overline{j}_{R_l^n} \Vert _{L^2(L_{R_l^n})} \left\| \frac{\partial (z_\mathbf{m} - z_\mathbf{m}^h)}{\partial t} \right\| _{L^2(L_{R_l^n})}\\&\quad+ \displaystyle \frac{1}{2} {\mathcal C} \Vert \overline{j}_{R_l^n} \Vert _{L^2(L_{R_l^n})} \big (h_l^n\big )^{\frac{1}{2}} \left( \displaystyle \max _{x \in K_l^n} L(x)\right) ^{\frac{1}{2}} \sum _{r=0}^{q}\, \sum _{j=1}^{m_n} \Vert \widetilde{z}_{j,r}^{\, n} - \widetilde{z}_{j,r}^{\, n, h} \Vert _{H^1(\widetilde{K}^n_l)} \Vert t^r\Vert _{L^2(I_n)}, \end{aligned}$$

where constant $\mathcal C$ depends on ${\mathcal C}_2$ in (35), *q* and $m_n$. We focus now on term $\mathrm{(III)}$ and, first of all, we apply again splitting (39):

$$\begin{aligned} \mathrm{(III)}&\le \left| \int _{{R_l^n}} {\mathcal J}_{n-1} \big [ z_\mathbf{m} - z_\mathbf{m}^h - T_n(z_\mathbf{m} - z_\mathbf{m}^h)\big ]^{n-1, +} \, d{R_l^n}\right| \\&\quad+ \left| \int _{R_l^n} {\mathcal J}_{n-1} \big [ T_n\big (z_\mathbf{m} - z_\mathbf{m}^h - {\mathcal I}^1(z_\mathbf{m}-z_\mathbf{m}^h)\big )\big ]^{n-1, +}\, dR_l^n\right| . \end{aligned}$$

Now, thanks to the mean value theorem, we remark that, for any function $w_\mathbf{m}\in V_\mathbf{m}^n$,

44 $$\begin{aligned} \big ( w_\mathbf{m} - T_n(w_\mathbf{m}) \big )^{n-1, +}= w_\mathbf{m}^{n-1, +} - w_\mathbf{m}(t_n^*)=-\int _{t_{n-1}}^{t_n^*} \displaystyle \frac{\partial w_\mathbf{m}}{\partial t}(s)\, ds \end{aligned}$$

with $t_n^*\in (t_{n-1}, t_n)$, as well as equality $\Vert {\mathcal J}_{n-1}\Vert _{L^2(S_{R_l^n})}=k_n^{\frac{1}{2}} \, \Vert {\mathcal J}_{n-1}\Vert _{L^2({R_l^n})}$ trivially holds. Moving from these results and by exploiting the definition of the projection operator $T_n$, the Cauchy–Schwarz inequality and estimate (40), we derive the final bound for $\mathrm{(III)}$:

$$\begin{aligned} \mathrm{(III)}&\le \left| \int _{S_{R_l^n}} {\mathcal J}_{n-1} \displaystyle \frac{\partial (z_\mathbf{m} - z_\mathbf{m}^h)}{\partial t} \, d{R_l^n} dt\right| \\&\quad+ \displaystyle \frac{1}{k_n}\left| \int _{S_{R_l^n}} {\mathcal J}_{n-1} ( z_\mathbf{m} - z_\mathbf{m}^h - {\mathcal I}^1(z_\mathbf{m}-z_\mathbf{m}^h)) \, dR_l^n dt\right| \\&\le k_n^{\frac{1}{2}}\, \big \Vert {\mathcal J}_{n-1} \big \Vert _{L^2(R_l^n)} \displaystyle \left\| \frac{\partial (z_\mathbf{m} - z_\mathbf{m}^h)}{\partial t} \right\| _{L^2(S_{R_l^n})}\\&\quad+ \frac{1}{k_n^{\frac{1}{2}}} \, \big \Vert {\mathcal J}_{n-1} \big \Vert _{L^2(R_l^n)} \big \Vert z_\mathbf{m} - z_\mathbf{m}^h - {\mathcal I}^1(z_\mathbf{m}-z_\mathbf{m}^h) \big \Vert _{L^2(S_{R_l^n})}\\&\le \big \Vert {\mathcal J}_{n-1} \big \Vert _{L^2(R_l^n)} \displaystyle \left\{ k_n^{\frac{1}{2}}\, \left\| \frac{\partial (z_\mathbf{m} - z_\mathbf{m}^h)}{\partial t} \right\| _{L^2(S_{R_l^n})} \right. \\&\quad+\left. \frac{{\mathcal C}}{k_n^{\frac{1}{2}}}\, \left( \displaystyle \max _{x \in K_l^n} L(x)\right) ^{\frac{1}{2}} h_l^n \, \sum _{r=0}^{q}\, \sum _{j=1}^{m_n} | \widetilde{z}_{j,r}^{\, n} - \widetilde{z}_{j,r}^{\, n, h} |_{H^1(\widetilde{K}^n_l)} \Vert t^r\Vert _{L^2(I_n)}\right\} , \end{aligned}$$

with $\mathcal C$ as in (41). The last term in (38) can be controlled by repeating the same computations adopted for $\mathrm{(III)}$, by replacing the temporal residual ${\mathcal J}_{n-1}$ with the initial error $e_\mathbf{m}^{h, 0, -}$ and by focusing on the first time interval. We achieve the following estimate

$$\begin{aligned} \mathrm{(IV)}&\le \big \Vert e_\mathbf{m}^{h, 0, -} \big \Vert _{L^2(R_{\widetilde{l}}^1)} \displaystyle \left\{ k_1^{\frac{1}{2}}\, \left\| \frac{\partial (z_\mathbf{m} - z_\mathbf{m}^h)}{\partial t} \right\| _{L^2(S_{R_{\widetilde{l}}^1})}\right. \\&\quad+\left. \frac{{\mathcal C}}{k_1^{\frac{1}{2}}}\, \left( \displaystyle \max _{x \in K_{\widetilde{l}}^1} L(x)\right) ^{\frac{1}{2}} h_{\widetilde{l}}^1 \, \sum _{r=0}^{q}\, \sum _{j=1}^{m_n} | \widetilde{z}_{j,r}^{\, 1} - \widetilde{z}_{j,r}^{\, 1, h} |_{H^1(\widetilde{K}^1_{\widetilde{l}})} \Vert t^r\Vert _{L^2(I_1)}\right\} , \end{aligned}$$

with $\mathcal C$ as in (43). Now, result (36) follows by properly combining the individual estimates obtained for terms $\mathrm{(I)}$–$\mathrm{(IV)}$. $\square$

Moving from (36), we propose as error estimator for the discretization contribution in (24) the value

45 $$\begin{aligned} \eta ^h=\sum _{n=1}^{N}\, \sum _{l=1}^{{\mathcal M}_n} \left[ \rho ^S_{R_l^n}(u_\mathbf{m}^h)\, \omega ^S_{R_l^n}(z_\mathbf{m} - z_\mathbf{m}^h) + \sum _{i=1}^2 \rho ^{Ti}_{R_l^n}(u_\mathbf{m}^h)\, \omega ^{Ti}_{R_l^n}(z_\mathbf{m} - z_\mathbf{m}^h) \right] , \end{aligned}$$

so that the estimator for the global functional error, $|J(\varepsilon _\mathbf{m}^h)|$, coincides with $\eta _{\mathbf{mm}^+}^h=\eta _{\mathbf{mm}^+}+\eta ^h$, with $\eta _{\mathbf{mm}^+}$ as in (19). In particular, since it is straightforward to distinguish in $\eta ^h$ the space from the time contribution given by

$$\begin{aligned} \eta ^h_S = \sum _{n=1}^{N}\, \sum _{l=1}^{{\mathcal M}_n} \rho ^S_{R_l^n}(u_\mathbf{m}^h)\, \omega ^S_{R_l^n}(z_\mathbf{m} - z_\mathbf{m}^h),\quad \eta ^h_T = \sum _{n=1}^{N}\, \sum _{l=1}^{{\mathcal M}_n} \sum _{i=1}^2 \rho ^{Ti}_{R_l^n}(u_\mathbf{m}^h)\, \omega ^{Ti}_{R_l^n}(z_\mathbf{m} - z_\mathbf{m}^h), \end{aligned}$$

respectively, it is immediate to decompose $\eta _{\mathbf{mm}^+}^h$ into a modeling, a space and a time contribution, as

46 $$\begin{aligned} \eta _{\mathbf{mm}^+}^h=\eta _{\mathbf{mm}^+}+\eta ^h_S+\eta ^h_T. \end{aligned}$$

This splitting will be crucial with a view to the global adaptive procedure. Both the estimators $\eta ^h_S$ and $\eta ^h_T$ share the structure characterizing a goal-oriented analysis, i.e., they coincide with the product of a residual depending on the primal solution and a weight related to the dual solution. In addition, we remark that, due to the HiMod procedure, the contribution along the *x*- and *y*-direction in the weights is split.

Some computational remarks on estimator $\eta ^h$ are now in order.

To make computable the weights, we replace the dual solution $z_\mathbf{m}$ with a computable discrete counterpart $z_\mathbf{m}^{*, h}$. A possibility is to resort to the discrete enriched dual solution $z_{\mathbf{m}^+}^h$. Nevertheless, since the temporal weights involve the time derivative of $z_\mathbf{m}$, we resort to a temporal recovery procedure yielding an approximation $z_\mathbf{m}^{*, h}$ that is at least linear in time. In particular, we follow the approach in [25, 26]. The dependence of the weights on the dual discretization error rather than on the dual solution is optimal in terms of convergence. Moreover, the time averaged residuals $\overline{r}_{R_l^n}$ and $\overline{j}_{R_l^n}$ make the estimator more reliable since $\Vert \overline{w} \Vert _{L^2(I_n)}\le \Vert w \Vert _{L^2(I_n)}$ as well as $\Vert w - \overline{w} \Vert _{L^2(I_n)}\le \Vert w \Vert _{L^2(I_n)}$, for any function $w\in L^2(I_n)$. An extra care has to be devoted to the computation of the temporal residual ${\mathcal J}_{n-1}$ that combines solutions associated with two different meshes. We use an interpolation operator from the degrees of freedom of ${\mathcal T}_{h_n}$ onto the ones associated with ${\mathcal T}_{h_{n+1}}$. Finally, the analysis in Proposition 3 may be generalized to a 3D framework provided that map $\psi _x$ is properly chosen. In particular, the orthonormality of basis $\mathcal B$ may be exploited to derive estimates (40) and (43) only if ${\mathcal D}^{-1}(x, \psi _x^{-1}(\widehat{\mathbf{y}}))$ does not depend on $\widehat{\mathbf{y}}$. This has to be explicitly demanded in a 3D setting while it always holds in a 2D framework.

### Building the space–time HiMod lookup diagram

Goal of this section is to keep the global functional error below a fixed tolerance TOL via an automatic selection of the modal distribution and now also of the space–time mesh $\big \{\big (K_l^n, I_n\big )_{l=1}^{{\mathcal M}_n}\big \}_{n=1}^N$.

**Fig. 12:**
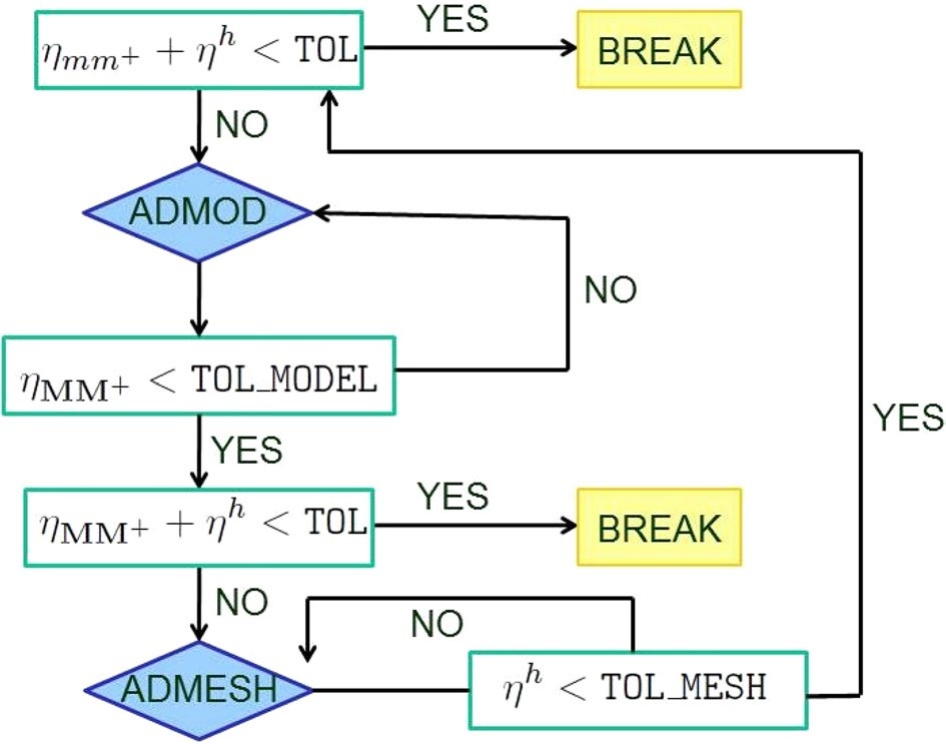
Flowchart of the global adaptive procedure

Different strategies are followed in the literature to combine model with mesh adaptation [11, 20, 21, 39]. The approach we propose iteratively alternates model with space–time mesh adaptation, by advantageously exploiting the additive structure of the global error estimator (46). For this reason, we distinguish a model (TOL_MODEL) and a mesh (TOL_MESH) tolerances, such that TOL_MODEL$+$TOL_MESH = TOL. Then, we follow the procedure outlined in Fig. 12. We distinguish two main modules, ADMOD devoted to model adaptation and ADMESH dealing with the space–time mesh adaptation. The module ADMOD exactly implements the five-stage adaptive procedure (S1)–(S5). Concerning the space–time mesh adaptation, the algorithm set by ADMESH is very straightforward, due to the one-dimensional nature of both the spatial and temporal meshes. In particular, while the space adaptation includes both mesh refinement (via bisection) and coarsening (gluing two consecutive intervals where $\eta _S^h$ is below tolerance), the time adaptive algorithm deals only with mesh refinement. This suggests to start the adaptive procedure on a sufficiently coarse time partition. Error equidistribution drives both the space and time adaptation. A maximum value constrains the number of iterations as well as tuning parameters $\delta _\mathrm{1H}$ ($=$0.5), $\delta _\mathrm{2H}$ ($=$1.5) limit the spatial mesh refinement and coarsening to the worst and to the best subintervals, respectively.

After a preliminary check on the accuracy of the global error estimator associated with the initial uniform modal distribution and the initial uniform space–time grid, model adaptation takes place till the accuracy TOL_MODEL is met by estimator $\eta _{\mathbf{MM}^+}$. Then, we check if model adaptation suffices to provide the global tolerance TOL without any space–time mesh adaptation. If not the module ADMESH is activated. In particular, we apply the spatial rather than temporal adaptation depending on which of the estimators $\eta _S^h$, $\eta _T^h$ is the greatest one. When $\eta ^h<$TOL_MESH, we come back to the initial check on the global accuracy.

A maximum number of iterations ensures the end of the whole adaptive procedure. We remark that each time the space–time partition is updated, a projection of the primal and dual solutions involved in the evaluation of the error estimator is demanded. As for the choice of the tolerances, we resort to a convex combination of the two tolerances, by selecting TOL_MODEL$=\theta$TOL and TOL_MESH$=(1-\theta )$TOL, with $0\le \theta \le 1$ [11]. The parameter $\theta$ settles a relation between model and discretization error, in accordance with requirement (22).

Finally, we refer to the outcome of the whole adaptive algorithm as to the space–time HiMod lookup diagram. Some instances of this table are provided in the next section.

### Numerical verification

In this section we assess the reliability of the global adaptive procedure.

### Reliability of the space–time adaptive HiMod reduction procedure

The test case used to validate the modeling adaptive procedure for $J=J_{\mathrm{mean}, T}$ is now tackled by activating the mesh adaptation as well. We preserve the same values of the previous run for TOL, for the initial uniform modal indices *m* and $m^+$, and for the initial space–time mesh. Then, we set $\theta =0.5$.

**Fig. 13:**
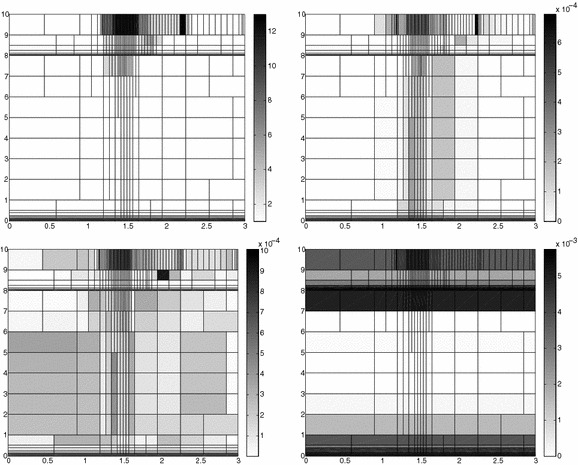
Convection–diffusion of a pollutant, control of $J_{\mathrm{mean}, T}$, global adaptation: space–time adaptive HiMod lookup diagram (*top-left*); space–time distribution of $\eta _{\mathbf{MM}^+}$ (*top-right*), of $\eta _S^h$ (*bottom-left*) and of $\eta _T^h$ (*bottom-right*)

**Fig. 14:**
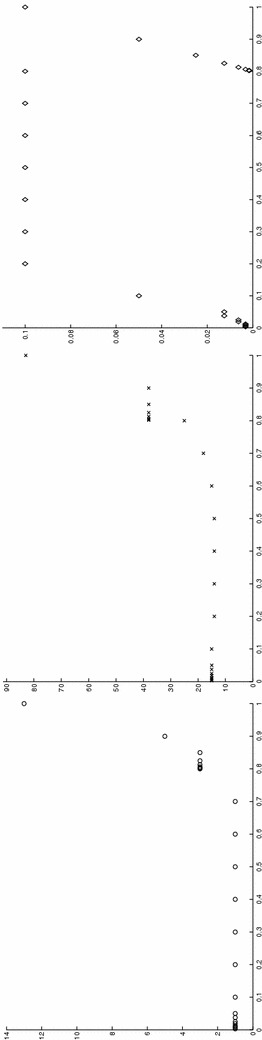
Convection–diffusion of a pollutant, control of $J_{\mathrm{mean}, T}$, global adaptation: modal distribution at x $=$ 1.5 as a function of time (*left*); mesh cardinality (*center*) and time-step (*right*) evolution

The adaptive procedure converges after 50 iterations, with 23 model iterations followed by nine and eight adaptations of the spatial and of the temporal mesh, respectively and by ten additional model adaptations. The final outcome of the adaptive procedure is the HiMod lookup diagram in Fig. 13, top-left. A comparison between this table and the one in Fig. 7, left shows a similar trend for the modes, i.e., a gradual increment of the number of modes as we approach the final time and in correspondence with the source location and the downstream areas. Nevertheless, the combination of model with mesh adaptation reduces from 3 to 1 the number of modes used in the first phase of the test case (compare Fig. 7, center with Fig. 14, left). Concerning the spatial adaptation, a coarse mesh consisting of less than 20 subintervals and refined around $x=1.5$ is predicted for the first time intervals. Then, this number increases with an abrupt variation in the last time interval when it reaches its maximum (see Fig. 14, center). The monotone trend characterizing the model and the spatial mesh adaptation is qualitatively the same, exhibiting a refinement of the modes and of the finite element partition confined to the last time intervals, in accordance with the goal quantity.

On the contrary, the time adaptation yields a non monotone prediction for the time step distribution, as depicted in Fig. 14, right. Essentially we recognize two phases when the initial time step is considerably reduced, the first one around the initial time and the second one just before time *T*. A strong refinement of the initial grid is recurrent in mesh adaptation and here it likely balances the initial rough modal and spatial discretizations. The second refinement occurs when the control of the mean value becomes more relevant. At time $t=0.8$, both the modal discretization and the space–time mesh are considerably refined to ensure the imposed tolerance. Probably, a complex interplay among the three discretizations takes place during the last time intervals, so that the severe demand on the time step can be then relaxed before reaching the final time.

**Fig. 15:**
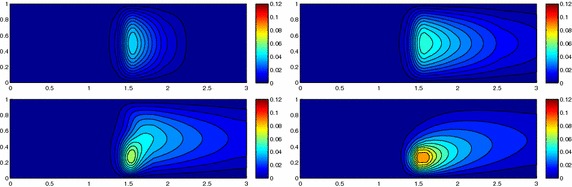
Convection–diffusion of a pollutant, control of $J_{\mathrm{mean}, T}$, global adaptation: HiMod approximation $u_\mathbf{M}^h$ at $t=0.1, 0.2, 0.8, 1$ (*top–bottom*, *left–right*)

Figure 13 gathers the distribution of the three error estimators on the space–time lookup diagram. The choice made for the tolerances leads to values of the same order of magnitude for $\eta _{\mathbf{MM}^+}$ and $\eta _S^h$, while the error estimator associated with the time discretization assumes larger values.

As shown in Fig. 15, the c[M($\mathbf{M}$)G(1)]-dG(0) HiMod solution associated with the diagram in Fig. 13, top-left is qualitatively different from the one in Fig. 6, right. The adoption of a single mode till $t=0.7$ identifies a reduced solution which is initially very far from the full one. Nevertheless, the time steps predicted by the adaptive algorithm are enough to refine, during the last time intervals, the number of modes as well as the partition along $\Omega _{1D}$ so that solution $u_\mathbf{M}^h$ becomes fully comparable with the full one at the final time.

**Fig. 16:**
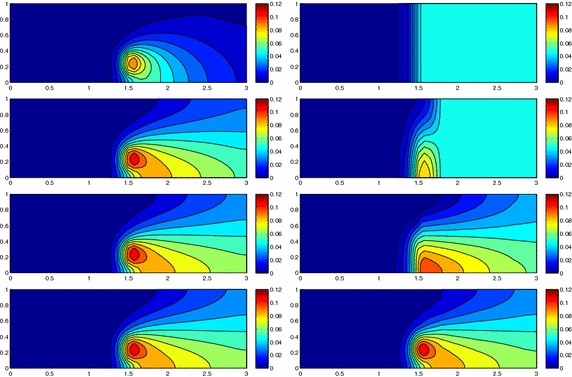
Convection–diffusion of a pollutant, control of $J_{\mathrm{mean}, T}^\mathrm{down}$, global adaptation: full solution (*left*) and pointwise HiMod approximation $u_\mathbf{M}^h$ (*right*), at $t=0.1, 0.5, 0.8, 1$ (*top–bottom*)

**Fig. 17:**
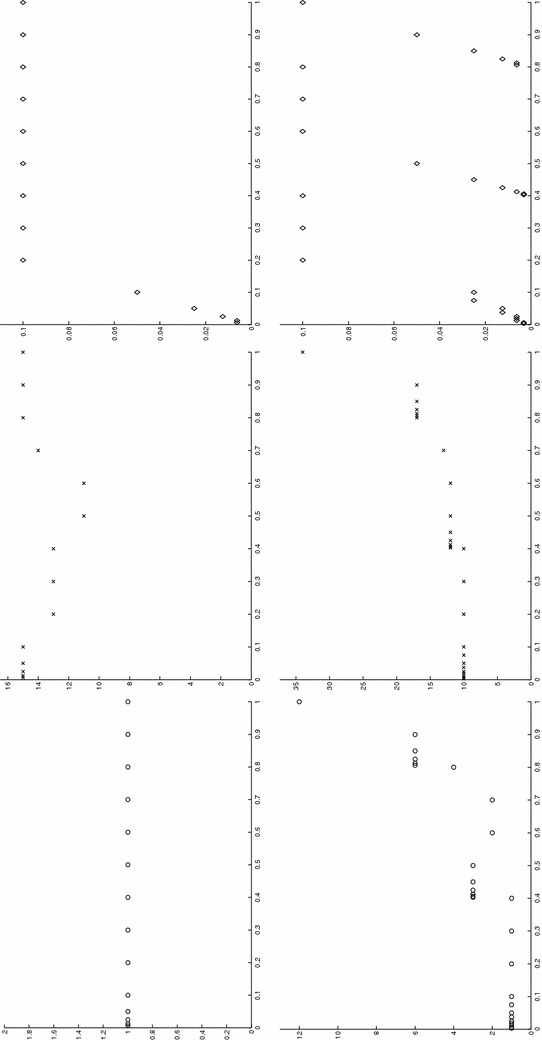
Convection–diffusion of a pollutant, global adaptation: space–time distribution of $\eta _{\mathbf{MM}^+}$ (*left*), of $\eta _S^h$ (*center*) and of $\eta _T^h$ (*right*), for $J_{\mathrm{mean}, T}$ (*top*) and $J_{\mathrm{mean}, T}^\mathrm{down}$ (*bottom*)

### Assignment of Neumann boundary conditions

We challenge the whole adaptive procedure by modifying the boundary conditions in the previous test case. We assign a homogeneous Neumann condition on the whole boundary, except for the edge $\Gamma _D=\{ (0, y):0\le y\le 1 \}$ where we preserve the homogeneous Dirichlet data. The new condition along the horizontal sides leads to select a new modal basis. After identifying the reference fiber $\widehat{\gamma }_1$ with the interval [0, 1], we choose ${\mathcal B}=\{\varphi _j(\widehat{y})= \sqrt{2}\cos (\pi j \widehat{y})\}_{j\in {\mathbb {N}}}$.

**Fig. 18:**
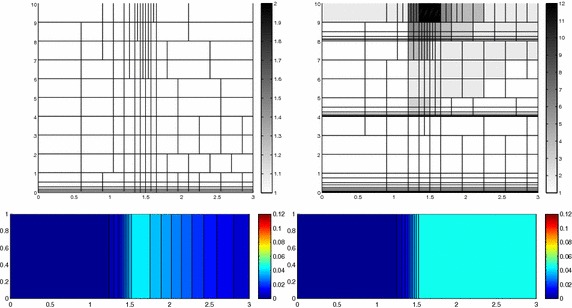
Convection–diffusion of a pollutant, global adaptation: space–time adaptive HiMod lookup diagram (*top*) associated with $J_{\mathrm{mean}, T}$ (*left*) and $J_{\mathrm{mean}, T}^\mathrm{down}$ (*right*); pointwise HiMod approximation associated with $J_{\mathrm{mean}, T}$ (*bottom*) at $t=0.2$ (*left*) and $t=1$ (*right*)

Figure 16, left shows the cG(1)-dG(0) full solution at four different times, computed with FreeFem++ on a uniform unstructured mesh of 10,252 elements. In particular, the new flux-free configuration erases the horizontal dynamics in Fig. 6, pushing the pollutant to contaminate also the northeast and the southeast areas. If we set the global adaptive procedure to control $J_{\mathrm{mean}, T}$, we do not expect much benefit from the modal basis since all the cosine functions have a null mean except $\varphi _0$. Figures 17, top and 18, top-left collect some results of the global adaptive procedure for TOL_MODEL$=$TOL_MESH$=5\times 10^{-3}$. The adaptive algorithm stops after ten iterations. No model adaptation is performed and only function $\varphi _0$ is switched on. On the contrary, both the spatial and the temporal meshes are adapted via seven and three iterations, respectively. The cardinality of the finite element mesh reaches a minimum in the middle of the interval *I*, while, after an initial refinement, the time step increases to the initial value 0.1. Overall, the modal-space–time discretization is coarse as shown by the HiMod lookup diagram. The correspoding c[M($\mathbf{M}$)G(1)]-dG(0) HiMod solution is provided in Fig. 18, bottom for two different times. It is not surprising that $u_\mathbf{M}^h$ looses the essential features of the full solution due to the deficiency of the reduced model. Smaller values of TOL, of course, do not modify this trend.

A completely different prediction is performed by selecting the goal functional $J_{\mathrm{mean}, T}^\mathrm{down}=[ \mathrm {meas}(\Omega ^\mathrm{down}) ]^{-1} \int _{\Omega ^\mathrm{down}} \zeta (x, y, 1)\, d\Omega ^\mathrm{down}$, with $\Omega ^\mathrm{down}=(0, 3)\times (0, 0.5)$. The global tolerance TOL$=10^{-2}$ is now guaranteed after 30 model iterations, followed by seven spatial and nine temporal mesh adaptations, plus a final model adaptation. The space–time adaptive HiMod lookup diagram yielded by the adaptive procedure is shown in Fig. 18, top-right. The number of cosine functions is gradually increased to eight in correspondence with $\mathcal D$. Additional modes are now demanded also upstream the source location in contrast to Fig. 13, top-left. The modal as well as the spatial mesh cardinality trend is very similar to the one in Fig. 14, whereas three refinements of the time step now occur (see Fig. 17, bottom). The additional refinement about in the middle of the time window corresponds to the phase when the pointwise HiMod solution starts to become similar to the full one. Indeed, as shown in Fig. 16, right solution $u_\mathbf{M}^h$ is initially far from the full one (and similar to the approximation in Fig. 18). Then, from $t=0.5$, $u_\mathbf{M}^h$ becomes more and more similar to the full solution till, at the final time, the two solutions are almost identical.

### Robustness of the space–time HiMod lookup diagram

**Fig. 19:**
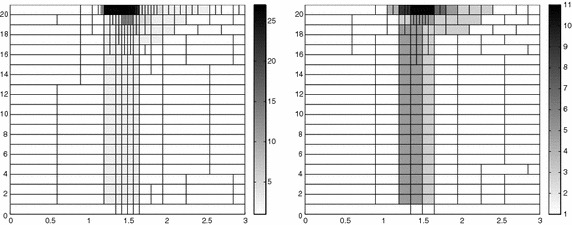
Robustness of the space–time HiMod lookup diagram: reference diagram (*left*); diagram yielded by the HiMod global adaptive algorithm (*right*)

**Fig. 20:**
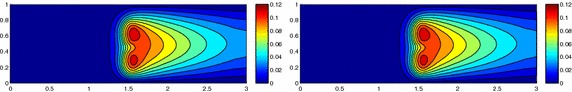
Robustness of the space–time HiMod lookup diagram: HiMod approximation based on the reference lookup diagram (*left*) and on the HiMod global adaptive algorithm (*right*)

We replicate the test performed for model adaptation, by checking the robustness of the space–time HiMod diagram with respect to possible variants of the reference problem. To this aim, we employ the space–time lookup diagram in Fig. 19, left tailored on the problem in Fig. 6 to build the HiMod approximation for the problem identified by the source term $f_3$ in Fig. 9, bottom. Figure 20 compares the approximation thus obtained (left) with the HiMod approximation yielded by the global adaptive procedure (right), whose space–time HiMod lookup diagram is provided in Fig. 19, right. The agreement between the two solutions is satisfying. The adaptive procedure optimizes the computational costs by predicting a lower number of modes and a coarser mesh. Nevertheless, the possibility of exploiting a previously computed HiMod diagram thus avoiding the cost of the adaptive procedure is a sufficient motivation to exploit the precomputed diagram.

### Computational saving

**Fig. 21:**
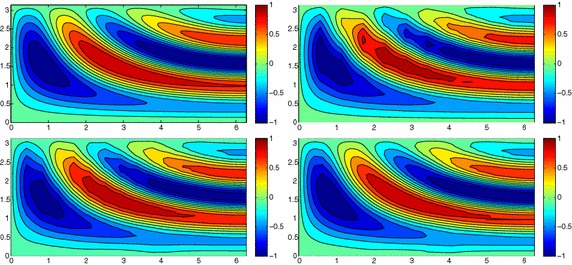
Computational saving check: full solution (*top-left*); uniform HiMod approximation for $m=17$ (*top-right*); pointwise HiMod approximations (*bottom*)

**Fig. 22:**
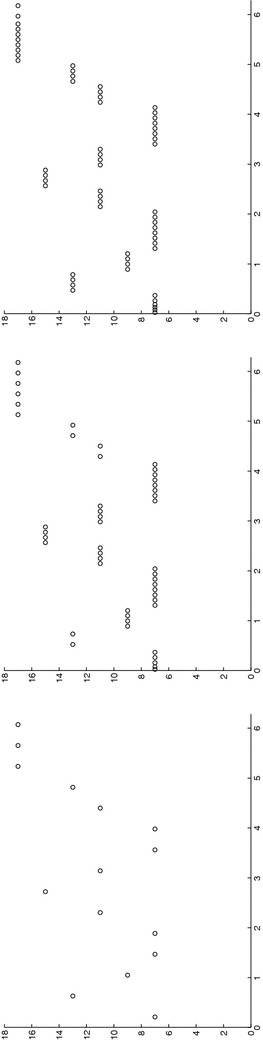
Computational saving check: modal distribution yielded by the modal (*left*) and by the global (*center*, *right*) adaptive procedure

Goal of this section is to verify the benefits due to the HiMod adaptation procedure in terms of CPU times^2^ with respect to a full and a uniform HiMod approximation. For the sake of simplicity, we consider a steady problem. We solve on the rectangular domain $\Omega = (0,2 \pi ) \times (0,\pi )$ the advection-diffusion problem $- \Delta u + \mathbf{c} \cdot \nabla u = f$, with $\mathbf{c}=(10, 0)'$, by assigning homogeneous Dirichlet data on $\partial \Omega \backslash \Gamma _N$, with $\Gamma _N=\{ (2\pi , y) :0\le y \le \pi \}$, and a homogeneous Neumann data on $\Gamma _N$. Then, we choose the source term such that the exact solution coincides with $u(x, y)=\sin y \sin \big ( 0.01 y (x^3 - 12 \pi ^2 x )\big )$ (see Fig. 21, top-left). We first investigate the advantages due to a uniform HiMod reduction with respect to a standard 2D finite element approximation. We fix a number of dof around 190 and we compute the $L^2(\Omega )$-norm of the error associated with the full approximation and with the uniform HiMod solution based on 17 modes and a uniform subdivision of the supporting fiber into 11 subintervals (see Fig. 21, top-right). As Table 1 shows, we gain an order of accuracy via the HiMod reduction. A comparison in terms of CPU time is not reasonable in such a case since the HiMod code is not yet optimized. By resorting to a modal adaptivity and for a comparable number of dof, we obtain a HiMod approximation more accurate with respect to the uniform one (compare the contour plots in Fig. 21, top-right and bottom-left and the values in Table 1) with a similar CPU time (in s). The modal distribution yielded by the adaptive procedure is shown in Fig. 22, left. A number of modes less than 17 is demanded on the whole domain except for the last three nodes. Concerning the CPU time, we quantify only the seconds demanded to build the HiMod approximation from the predicted modal distribution, since we have verified the robustness of the HiMod diagrams.

We now add the adaptivity of the spatial mesh. Table 2 compares the accuracy of a full with a HiMod approximation for about the same number of dof. We adopt two different tolerances to drive the global adaptive procedure. The corresponding HiMod diagrams are shown in Fig. 22, center and right. The accuracy characterizing the adapted HiMod solution is higher in both the cases and the computational times remain contained. Figure 21, bottom-right shows the HiMod approximation characterized by 622 dof. The maximum number of modes predicted by the adaptive procedure is still 17 but it is evident that the employment of an adapted mesh improves the reliability of the reduced solution as it is qualitatively evident by comparing the two contourplots in Fig. 21, bottom.

**Table 1:** Computational saving check: comparison between full and HiMod approximations for about the same number of dof

Full		Uniform HiMod			Model adaptation		
dof	Error	dof	Error	CPU time	dof	Error	CPU time
190	0.506	187	0.084	0.453	193	0.045	0.407

**Table 2:** Computational saving check: comparison between full and HiMod approximations for about the same number of dof

Full		Model $+$ mesh adaptation		
dof	Error	dof	Error	CPU time
630	0.148	622	0.027	1.321
989	0.091	966	0.018	2.035

## Validation of the HiMod reduction

This is a first attempt of validation for the HiMod reduction procedure. For this purpose, we focus on the experimental and modeling analysis provided in [27] dealing with a reactive transport in homogeneous porous media.

**Fig. 23:**
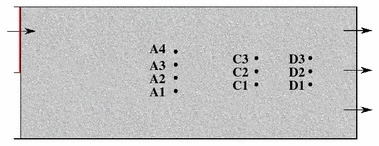
Diagram of the experimental configuration used for the validation

We consider the experimental setting outlined in Fig. 23. It consists of a rectangular laboratory flow cell of dimension 2.5 dm $\times 1$ dm $\times 0.08$ dm along the *x*-, *y*- and *z*-direction, respectively. The cell is filled with a porous media with measured porosity equal to 0.375 and it is initially saturated with an aqueous solution. Segment $\Gamma _\mathrm{inlet}=\{ (0, y, z):0.5\le y \le 1, 0\le z \le 0.08\}$ coincides with an inlet boundary, where a constant concentration, modeling the injection of a reactive component, is assigned. Simultaneously, a flow rate of 12 ml/h is set at the outlet $\Gamma _\mathrm{outlet}=\{ (2.5, y, z):0\le y \le 1, 0\le z \le 0.08\}$, resulting in an average water velocity of about 0.404 dm/h at the equilibrium. We remark that the set-up of the experiment is designed to have a pseudo-1D flow, parallel to the *x*-axis. Finally, ten sampling ports are located in the cell, to collect measurements of the reactive fluid concentration. Sampling is performed four times during each experiment. The concentration measurements represent the data we aim at matching via a HiMod reduced modeling in the same spirit of the analysis in [27]. The reactive transport experiment is conducted for 60 h, though a stationary state is reached already after 15 h from the beginning of the experiment, so that we restrict the time window of investigation to (0, 30).

For all the further experimental data we refer to [27] since a greater level of detail on the experimental setting is beyond the purposes of the paper.

**Fig. 24:**
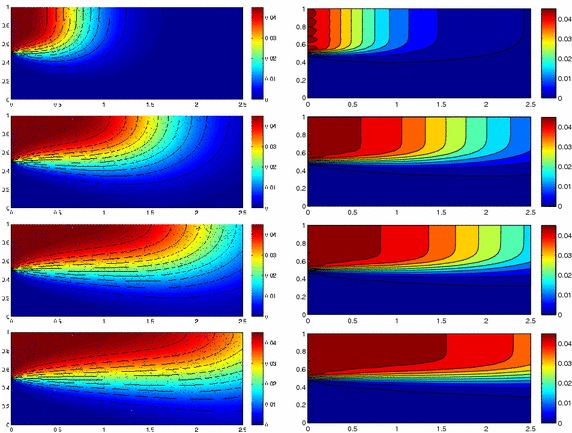
Reactive transport in porous media: full solution (*left*) and uniform HiMod approximation $u_{20}^h$ (*right*) at $t=5$, 11, 15, 19 h (*top–bottom*)

From a modeling viewpoint, since the setting is invariant along the *z*-axis, we can simulate the experiment in an effective way as a two-dimensional flow. In particular, we adopt the unsteady equation

47 $$\begin{aligned} \left\{ \begin{array}{ll} \displaystyle \frac{\partial u}{\partial t}(x, y, t) - 0.00085\,\Delta u(x, y, t) + 0.404 \, \frac{\partial u}{\partial x}(x, y, t) = 0 &{}\quad (x, y, t)\in \Omega \times (0,30)\ \\ \displaystyle \frac{\partial u}{\partial y} (x, 0, t) = \frac{\partial u}{\partial y} (x, 1, t) = 0 &{}\quad 0\le x < 2.5,\ t\in (0,30)\\ u(0, y, t) = 0 &{}\quad 0\le y < 0.5,\ t\in (0,30)\\ u(0, y, t) = 0.045 &{}\quad 0.5\le y < 1,\ t\in (0,30)\\ \displaystyle \frac{\partial u}{\partial x} (3, y, t) = 0 &{}\quad 0\le y < 1,\ t\in (0,30)\\ u(x, y, 0) = 0 &{}\quad (x,y) \in \Omega , \end{array} \right. \end{aligned}$$

with $\Omega =(0, 2.5)\times (0, 1)$, to model the process of advection and diffusion of the reactive component. Notice that (47) represents a simplified version of the original model in [27]. A preliminary tuning of the model parameters has been carried out to make the solution of the two models as close as possible in the considered experimental context. In more detail, we adopt a constant diffusive coefficient whose value is set, via a trial and error procedure, to replicate the action of the diffusive tensor used in [27]. Moreover, following [27], we select the value for the flux velocity by solving an additional Darcy problem.

**Fig. 25:**
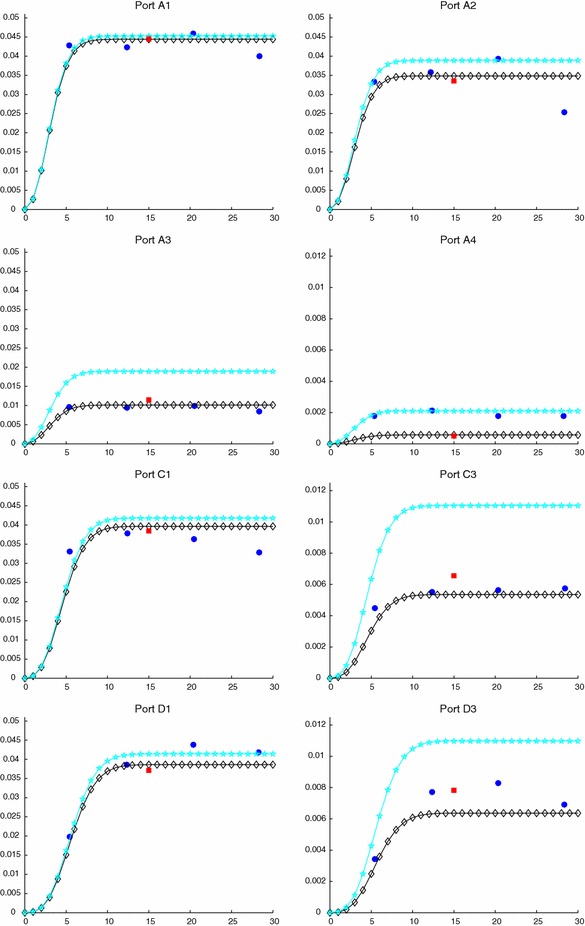
Reactive fluid concentrations at the sampling ports *A1, A2, A3, A4, C1, C3, D1, D3* (*top–bottom*, *left–right*): measured (*circle symbols*) and simulated concentrations via the full finite element discretization (*star symbols*), $u_{20}^h$ (*diamond symbols*) and $u_\mathbf{M}^h$ (*square symbols*)

Figure 24, left shows the full solution computed with FreeFem++ on a uniform unstructured mesh of 13,078 triangles at $t=5$, 11, 15, 19 h. The reactive fluid gradually spreads into the flow cell.

We now test the HiMod reduction procedure. We first resort to a uniform HiMod approximation and we use 20 modal functions to describe the transverse dynamics. We adopt a uniform space–time discretization along $\Omega _{1D}$ and (0, 30), with step $h=0.05$ and $k=0.5$, respectively. In Fig. 24, right we gather the HiMod solution $u_{20}^h$ at $t=5$, 11, 15, 19 h. The reliability of the reduced solution is satisfactory, despite the considerable reduction of the (spatial) dof (1000 vs 13,078). Now, we focus on the actual validation phase. For this purpose, in Fig. 25, we compare the measured (circle symbols) with the simulated concentrations (diamond symbols for the uniform HiMod approximation and star symbols for the 2D finite element discretization) in correspondence with eight of the ten sampling ports in Fig. 23. We refer only to one of the two sets of data available in [27]. Qualitatively, at each port, we recognize a first phase of about 8 h when the chemical breakthrough, characterized by a sigmoid shape curve, occurs; successively, the steady state is reached and each curve exhibits a plateau. The agreement between simulated and measured concentrations is good and comparable with the one of Fig. 3 in [27]. In particular, the full approximation improves the tracking of the data in correspondence with port A4. On the contrary, the prediction at ports A3, C3, D3 is more reliable when resorting to the HiMod approximation, despite the reduced number of dof.

**Fig. 26:**
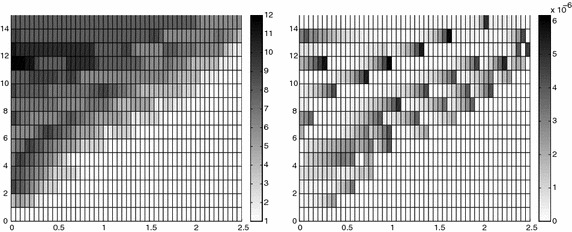
Reactive transport in porous media, control of $J_{15}$, modal adaptation: HiMod lookup diagram (*left*) and corresponding space–time distribution of $\eta _{\mathbf{MM}}^+$ (*right*)

As last test, we assess the reliability of the modeling adaptive procedure in a validation context. We aim at evaluating the reactive fluid concentration at $\widetilde{t}=15$ h via the c[M($\mathbf{M}$)G(1)]-dG(0) HiMod solution predicted by the modeling adaptive procedure. We consequently choose functional *J* as $J_{15}(\zeta )=[ \mathrm {meas}(\Omega ) ]^{-1}\int _{\Omega } \zeta (x, y, 15)\, d\Omega$. The expectation is to obtain a value for the concentration similar to the one provided by $u_{20}^h$ and not so far from the experimental data. We set the adaptive algorithm with TOL$=10^{-3}$, $m=1$, $m^+=3$. Concerning the space–time discretization, we fix a uniform space–time subdivision of $\Omega _{1D}\times I$, with $h=0.05$ and $k=0.5$. Finally, we reduce the time window to (0, 15) due to the stationary regime of the flow in the interval (15, 30).

**Fig. 27:**
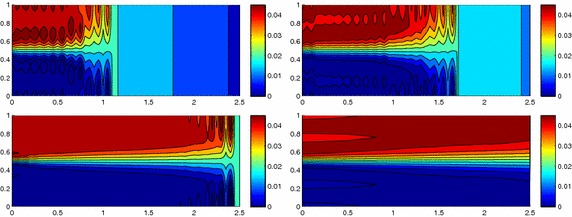
Reactive transport in porous media, control of $J_{15}$, modal adaptation: HiMod approximation at $t=5$, 7, 11, 15 h (*top–bottom*, *left–right*)

The modeling adaptive algorithm converges after 599 iterations and provides the HiMod lookup diagram in Fig. 26, left characterized by the space–time distribution of $\eta _{\mathbf{MM}^+}$ in Fig. 26, right. Both the diagrams corroborate the complexity of this experiment. In contrast to a more localized phenomenon such as the convection–diffusion of a pollutant in the previous sections, the refinement of the number of modes now gradually involves the whole $\Omega _{1D}$ as we approach time $\widetilde{t}$. The non uniform trend of the estimator highlights the demanding work performed by the adaptive procedure to guarantee tolerance TOL. Despite these difficulties, the maximum number of modal functions required by the lookup diagram is 12 to be associated with the area closer to the inlet and with the time intervals immediately preceding the steady state. The pointwise HiMod approximation $u_\mathbf{M}^h$ generated by the online phase is depicted in Fig. 27, for t $=5$, 7, 11, 15 h. The trend of the adapted solution becomes more and more similar to the one in Fig. 24, as *t* approaches $\widetilde{t}$.

Finally, we examine the concentration values predicted by the adapted HiMod solution at $\widetilde{t}=15$ h in correspondence with the eight ports in Fig. 25 (see the square symbols). It is evident the good matching of the simulated concentrations between $u_{20}^h$ and $u_\mathbf{M}^h$, with a slight different prediction at ports C3 and D3.

## Conclusions and perspectives

We have successfully extended the pointwise HiMod approach to an unsteady setting, by formalizing the so-called c[M(**M**)G(s)]-dG(q) HiMod reduction procedure. The goal-oriented *a posteriori* error analysis has allowed us to devise an automatic algorithm to select the reduced model, that guarantees the desired accuracy on the functional of interest. The results yielded by the global adaptive procedure are very satisfying, despite the complex interplay among the three adaptations. The sensitivity of the predicted HiMod reduced model with respect to the goal quantity and the assigned boundary conditions has been correctly validated. We have also verified the robustness of the HiMod lookup diagrams, by showing that, although strictly tailored to the problem at hand, they can be employed to deal with certain variants of such a problem. The computational advantages guaranteed by a HiMod reduction have been checked as well. Finally, the preliminary validation results in the last section are absolutely promising with a view to an effective application of HiMod to practical problems.

Prospective extensions of HiMod reduction include the approximation of nonlinear as well as 3D problems. This will be a crucial effort with a view to our last goal, i.e., to use HiMod reduction for the simulation of the blood flow in the arterial system.

## Footnotes

1 To simplify notations, with the super-index *h* we understand both the space and time discretizations.

2 All the experiments have been performed using Matlab 2011a 64-bit on a Lenovo ThinkPad T430 equipped with a Intel Core i5 3320M 2x 2.6–3.3 GHz processor and 4 GB of RAM.
